# Supplementary material for: Implementing essential diagnostics-learning from essential medicines: A scoping review
Source: PLOS Glob Public Health. 2022 Dec 19;2(12):e0000827. doi: 10.1371/journal.pgph.0000827 (PMC10121180; doi:10.1371/journal.pgph.0000827)
Supplement: S1 Table — (DOCX) [file pgph.0000827.s002.docx]

**Barriers to the implementation of Essential In Vitro Diagnostics List (EDL)**

| **Subthemes** | **Codes** | **Illustrative quotes** |
| --- | --- | --- |
| **Theme: Level – Health system constraints** | | |
| Accessibility of care | Poor financial access   - Out of pocket expenses | Uninsured, or if the facility does not or cannot accept insurance, there is an out-of-pocket price for the patient. |
|  | Poor financial access   - High test costs | The biochemical test price is the lowest in each hospital. The haematology and microbiology test prices are about three times as high. The histopathology test price is about 15 times as high as the price of the glucose test, using the median relative price of the five hospitals. |
|  | Poor geographical access   - Poor rural access to EDL | Compared to rural labs, an urban lab had a trending association with higher EDL availability. |
|  |  | A higher availability in urban facilities compared to rural facilities. |
|  | Poor health facility access   - Poor access to tests in public hospitals | Access to diagnostic test and devices were low in a public hospital. |
|  |  | A greater variety of tests in the private sector compared to the public sector. |
|  | Poor health facility access   - Poor access in lower-level health facilities | The lower level of facilities had low availability of EDL tests. |
|  |  | Primary care unit levels had significant gaps in essential diagnostic availability. |
| Financial resources | Inadequate health facility operational funds | 14 facilities identified financial constraints. |
|  | High test costs | Pricing of tests depends on the diagnostics test, with histopathology costing more. |
| Human resources | Shortage of staffs | Reported shortage of staff limited provision of laboratory tests. |
|  | Inadequate capacity | Laboratory technicians were adequate only in 25% (5/20) of hospitals. |
| Education system | Improper training | Improper training in providing laboratory tests was reported. |
|  | Lack of training | Health workers felt that knowledge gaps led to delayed identification of cases and management practices that deviated from accepted guidelines. |
| Information systems | Unavailability of operational data | Data on the accessibility of essential in vitro diagnostics in community-based health planning and services sites. 4 of the 12 supported districts have missing data for more than 6 consecutive weeks. |
| Facilities | Unavailability of tests | The availability of EDL tests is low. |
|  |  | All primary care unit levels had significant gaps in essential diagnostic availability. |
|  |  | No rapid tests were available (urine, blood glucose or HbA1c) for prompt risk stratification. |
|  |  | The only laboratory test uniformly available at all surveyed facilities was random blood glucose. |
|  |  | There is only one mention of TB diagnosis among the top 25 tests in UCH Ibadan. |
|  |  | 57.2% of Primary Health Care Unit level reported stock problems. |
|  |  | Some of the tests in the WHO EDL were not available in any of the health centres, such as whole blood lactate, HIV self-testing and sputum LAMP. |
|  |  | Inadequate levels of HIV Rapid Diagnostic Tests (RDTs |
|  |  | Occasional stockouts were registered for cholesterol and uric acid. |
|  |  | The low levels of Uni-Gold stock experienced across our study facilities. |
|  | Reagent stock-outs | Low availability of key consumables for laboratory diagnosis. |
|  |  | Reagent stockouts intermittently disrupted services at Mulago, Mbagathi, Migori, and Queen Elizabeth Central Hospitals. |
|  | Lack of proper equipment and supplies | Facilities faced a lack of proper equipment and supplies. |
|  |  | Limited items of the major laboratory equipment. |
|  |  | The most widespread challenge, reported by 44 facilities, was the lack of proper equipment and supplies. |
|  |  | Limited items of the major laboratory equipment and stock problems in maintaining main supplies for essential laboratory services provision. |
|  | Inadequate infrastructure | Issues with general infrastructure and space were mentioned by 22 facilities, with 7 describing problems with electricity. |
|  |  | Rural facilities had inadequate accommodations for their staff, who commuted for days limiting the provision of laboratory tests. |
| Procurement and distribution systems | Supply chain gaps | Supply chain management issues poor forecasting and low inventory levels |
| Relationship with norms and standards | Poor availability of guidelines | The Society for Endocrinology, Metabolism and Diabetes of South Africa (SEMDSA) guidelines were unavailable in any clinic. |
| **Theme: Level – Social and Political constraints** | | |
| Legislation or regulations | Insufficient policy | The lack of articulated policies concerning Rheumatic heart disease (RHD) contributes to poor funding for RHD services and diagnosis. |

**Barriers to the implementation of the Essential Medicines Lists (EML)**

| **Subthemes** | **Codes** | **Illustrative quotes** |
| --- | --- | --- |
| **Theme 1: Level – Recipient of care constraints** | | |
| Knowledge and skills | Inadequate knowledge | This is attributed to parents' inadequate knowledge of diseases. |
|  |  | However, retail consumers lack both the medical and technical information to judge their purchase of products. |
|  |  | Patients provided their reason for not seeking care, including no drugs available in the area. |
| Attitudes regarding programmes acceptability, appropriateness, and credibility. | Poor financial access | Participants perceived that consultation and drugs were too expensive. |
|  | Low health-seeking behaviour | Patients reported not seeking care because the health problem was not considered severe. |
|  |  | According to SARA service availability was low due to low health-seeking behaviour. |
|  | Uncertainty of services   - Unmet needs/services | Clients refused referrals for logistic and service responsiveness reasons such as the uncertainty of the kind of reception they would get at the receiving hospital. |
|  |  | Participants believed that women undergo an unnecessary cesarean section in some health facilities, and thus women avoid giving birth in these facilities. |
|  | Medicine quality and safety concerns | Five CHMT members in Mkuranga had observed adverse effects in clients taking sulphadoxine-pyrimethamine (SP) and expressed some concern about its safety. |
|  |  | Consumers felt that generic medicines were inferior and referred to these as" fong kong", a term used to describe fake goods in Johannesburg. Cheaper medicines were considered inferior, and people viewed these with a degree of suspicion |
|  | Alternative services | The Percent of patients who believe that the private pharmacy's medicine is better than the primary health care centre (PHC) was significantly higher in rural than urban groups (24% and 10%, respectively. |
|  |  | 30% of the patients in the rural unit believed that herbal treatment is better than drugs compared to 22.5% in the urban unit. |
|  |  | Most of the respondents preferred the patent medicine vendors and pharmacies (53.63 %) as the primary source of care. Only 7.6% utilize the PHC centres in the villages; others use traditional medicine (16.7%) and private health facilities (14%) |
|  | Social-cultural influences | Socio-cultural influences were also highlighted as fuelling stigma and the reason some ART patients chose to go to facilities further away from their homes rather than nearer facilities. |
|  |  | Mothers' attitudes and beliefs were perceived as barriers to facility delivery in this study. Men also perceived home delivery as safe in the rural community, and women were convinced to deliver at their home |
| Motivation to change or adopt a new behaviour | Low availability of drugs | Patients provided their reason for not seeking care, including no drugs available in the area. |
|  | Poor financial access   - Unaffordable medicines | In this study, 63.9% of the patients characterized dispensed medicines as not affordable. |
|  |  | Most of the patients (94.3%) could not afford their medicines. |
|  | Poor health access   - Poor accessibility to facility | Sudden onset of labour and labour starting at night were the major reasons perceived for home delivery |
|  | Poor recipient of care and health workers' relations | Negative attitudes of some nurses towards pregnant women can act as a deterrent to expectant mothers. |
|  |  | The negative attitude of staff was the main reason among 8.0%; only 0.3% mentioned the absence of a medical doctor as their reason for not utilizing the primary health care facilities. |
|  | Lack of social support | Lack of husband support, lack of women's autonomy in decision-making concerning the use of maternal healthcare services and stigma on unmarried teenage pregnancy were perceived as barriers to accessing medical care |
|  |  | Lack of welfare support and the household financial condition play a vital role in women's access to health services since over 50% of the country's population live below the poverty line. |
|  |  | Poor community involvement accounted for 9.2%. |
|  | Inadequate supplies | Coping with medical equipment shortages, pregnant mothers at one health centre were asked to bring gloves, typically during labour and delivery, to prevent the possibility of infection. |
| **Theme 2: Level – Providers of Care constraints** | | |
| Knowledge and skills | Inadequate providers' knowledge | 137 (42.7%) mothers were satisfied with the knowledge/competency of HEWs |
|  |  | About half of the health care workers (51%) interviewed were knowledgeable of the Integrated Management of Childhood Illness (IMCI) approach. |
|  |  | Over 40% of non-bacteria diarrhoea in children received antibiotics, which are not in line with the standard treatment guidelines (STG). |
|  |  | Providers poorly understood the meaning of palliative care. |
|  |  | Of the knowledgeable proportion, respondents in only 47%; 122 outlets (121 drug shops and one pharmacy) indicated that they knew about a potential source and could be able to obtain loans from financial credit institutions. |
|  |  | This limited understanding of the child-appropriate dosage formulations by key stakeholders was a barrier to their integration into the health policy. |
|  |  | Lack of knowledge of basic expiry prevention tools. Not knowledgeable about (first expiry, first-out) FEFO and (first in, first out) FIFO inventory management. |
|  |  | Not aware of the Novartis access programme (which aims to increase availability and reduce prices for essential medicines). |
|  |  | The Society for Endocrinology, Metabolism and Diabetes of South Africa (SEMDSA) guidelines were unknown. |
|  | Inadequate capacity | Data from the eight sub-district health facilities indicated shortages of skilled healthcare staff were a persistent challenge to healthcare management.  Three-quarters of the sub-district healthcare settings did not have the necessary skilled staff to manage the obstetric case. |
|  |  | There are inadequate levels of skilled birth attendants. |
|  |  | (Patient medicine vendors) PMVs lack the requisite pharmaceutical training and qualifications. |
|  |  | In all health facilities, the responsible person for managing priority medicines was a pharmacy technician, and 9 (90%) did not receive training in logistics. |
|  |  | None of the doctors was skilled in performing emergency hysterectomies during and after a caesarean section. |
|  | Varying treatment approaches | Different physicians have different treatment approaches. |
|  | Unequitable training   - Geographical access | A higher proportion of health care workers received management malaria, pneumonia, and diarrhoea in urban areas compared to rural areas. |
|  | Inadequate training | The availability of health providers working in antenatal care who had received technical updates in ANC over the last two years was low. |
|  |  | Very few providers had received recent antenatal care (ANC) training. |
|  |  | Few referral district hospitals (CHD) providers had received comprehensive emergency newborn care (CEmONC) technical updates. |
|  |  | The results also indicate that although many Patient medicine vendors (PPMVs) stock treatments for pneumonia, diarrhoea, and malaria, knowledge of the most effective treatment is lower. |
|  |  | Among Patient medicine vendors (PPMVs) without health training, 67.7 % had completed an apprenticeship; 32.3 % were current PPMV apprentices or did not report any form of formal or informal training. |
|  |  | Across all basic health facilities surveyed, no staff had been trained in Emergency Obstetric & Newborn Care (EmONC) in the preceding two years. |
|  |  | Several health care workers reported a lack of in-service training. |
|  |  | Nurse ward managers lacked specific training in quality improvement, and they were also worried about the knowledge deficit in this critical area of their managerial role |
|  |  | Some of the midwives interviewed reported that they had been left out during the initial Integrated Management of Childhood Illness (IMCI)training, perhaps because it was never anticipated that they would ever interface with children. |
| Attitudes regarding programmes acceptability, appropriateness, and credibility. | Quality concerns of medicines | Some providers had negative perceptions about products supplied by companies from India and China. |
|  | Concerns about adopted ICT systems | In some hospitals, staff also lacked confidence in the use of RxSolution. As a result, its use was minimal.  Storeroom staff did not count and verify stock physically since the quantities produced by the software were not always reliable. |
|  | Alternative medicine | Findings on the attitude of health workers towards traditional medicine showed that 50% of the respondents knew Traditional medicine they considered it effective on other illnesses. |
|  | Negative attitudes | Nurse managers reported negative staff attitudes toward new quality improvement projects to improve the quality of care. |
|  |  | Poor perceptions and awareness of child-appropriate medicine dosage formulations. |
|  |  | Limited prescription of pain medication was further exacerbated by providers‚ tendency to stigmatize palliative care as end-of-life care |
| Motivation to change or adopt a new behaviour | Low level of motivation   - Low interest - High workload - Limited career opportunities - Limited training opportunities | Most caregivers have low interest in working in delivery rooms. |
|  |  | Absenteeism was associated with a low motivation to work due to the lack of essential medicines and equipment for nurses to use in the ward. |
|  |  | Underpayment of health workers contributes to poor motivation amongst health workers. |
|  |  | Delays in the payment of claimant rebates to health staff and the demotivation of health professionals. |
|  |  | Low morale and industrial actions by health workers. |
|  |  | The excessive workload was one of the major reasons health workers might resist participating in the nurse-initiated management of the antiretroviral treatment (NIM-ART) programme. |
|  |  | Limited career opportunities |
|  |  | Insufficient supervision and training opportunities. |
|  | Limited evidence approached decision making | Some informants disagreed with such evidence, which indicated how difficult it was for scientific evidence to find its way onto the decision-making table compared with clinical experience. |
| **Theme 3: Level – Other stakeholders (community health committees, community leaders, programme managers, donors, policymakers, opinion leaders** | | |
| Knowledge and skills | Inadequate knowledge | Some of the Health Facility Governing Committee (HFGC) members felt that they did not have much knowledge on issues related to health commodities and financial reports. |
|  |  | It was evident in the study that stakeholders have varying levels of understanding of the concept of child-appropriate dosage formulations. |
|  | Unequal access to information | Asymmetric information favours sellers, generating incentives for cheating, such as selling sub-standard and inappropriate products and unprofessional behaviour, such as over-prescribing. |
|  | Inadequate training | There is irrational use of medicine exacerbated by a lack of sufficient training of prescribers in the hospital. |
| Motivation to change or adopt a new behaviour | Low level of motivation | Only 37% of participants agreed that they were proud to be part of the Ideal Clinic Realisation and Maintenance (ICRM) programme. |
|  | Inadequate medical supplies | Providers felt that mobile services provided inadequate access to medication for patients with comorbidities. They collected part of their medication from the mobile clinic and travelled to the facility to collect their chronic medicines. |
|  |  | The lack of drugs and medical supplies created a challenging working environment for health care providers. Respondents reported disappointment because they felt they were providing incomplete care. |
|  | Poor health worker and recipient of care relationship | Fear of disrespect and abuse by health care providers. Verbal abuse in the form of shouting or rudeness, and physical abuse in the form of pinching. |
| **Theme 4: Level – Health system constraints** | | |
| Accessibility of care | Poor geographical access   - Lack of transport - Unavailability of staff in PHCs - Long-distance to health facilities - Transport costs to health facilities | The geographical terrain, the region where this hospital was located, had been dealing with a civil war for many years. The region also had a poor road network that often made it inaccessible |
|  |  | Lack of transport prevents result in women from giving birth at home. At night there were reports of PHCs not being open or staff not being available. |
|  |  | All the men and many women who participated in the study perceived poor condition of the roads, remoteness, lack of transport, and long distance to the nearest health facilities as the major obstacles for women reaching a health facility for delivery or when they developed complications. |
|  |  | In a few cases, the urban availability of each tracer medicine was consistently above or equal to rural availability. |
|  |  | Rural private facilities were more likely to be drug shops than urban facilities. |
|  |  | Due to the distance to the health facility, women are forced to deliver at home. |
|  |  | Participants travelled for approximately 30 mins -1 hour to the nearest healthcare facility. |
|  |  | Rural areas had more difficulties with access than urban areas owing to longer distances and lesser facilities. |
|  |  | Patients often must travel far to access medicines, costing money. |
|  |  | Lack of transport due to long distances to the facilities, long waiting times, sharing of service delivery points with other patients, and late opening of the clinic. |
|  | Poor equitable access   - Marginalized groups had greater difficulty accessing care | Marginalized groups had greater difficulty accessing care and would experience harsher treatment from providers, preventing them from returning for additional care. |
|  | Poor financial access   - High costs - Out of pocket costs - Higher medicine costs in private facilities | Substantial changes in price from week to week introduce uncertainty for patients and limit access. |
|  |  | Most of the patients (94.3%) could not afford their medicines. |
|  |  | The essential medicines are not affordable. |
|  |  | Antibiotics were more expensive in private pharmacies, as also reported elsewhere. |
|  |  | The costs of tests and medicines are largely unaffordable. |
|  |  | Costs could be incurred through direct fees and more indirect costs such as buying drugs and other supplies or other hidden charges. |
|  |  | Caretakers reported not purchasing the drugs prescribed, mainly because they could not afford them. |
|  |  | The high cost of medicines was highlighted by participants irrespective of their socio-economic level. |
|  |  | Internationally Controlled Essential Medicines (ICEMs) were also not always available in the public sector, and all medicines are free to the patient, forcing patients to seek care from the private sector, where costs are substantial. |
|  |  | High cost of services such as paying for the glucose check and buying medicines due to stock-outs in public facilities. |
|  |  | One-third of patients who did not get the prescribed drugs could not pay for the medicine. |
|  |  | The COVID-19 pandemic affected the cost of essential medicines for both acute and chronic illnesses, as 75.0% of those with acute illnesses and 74.0% of those with chronic illnesses attested to an increase in the cost of medicines during the pandemic. Altogether, 77.3% of the respondents observed a rise in medical costs. |
|  |  | Drug outlets and private non-profit facilities charged higher prices than public facilities. |
|  |  | Patient prices were 36 % times higher in the private sector than in the public sector.  Medicines were unaffordable for treating common conditions prevalent in the zone in both public and private sectors.  Lowest priced medicines are unaffordable for 70 % of standard treatments of prevalent infectious diseases in both sectors. |
|  |  | However, we found that people in poorer areas were so often paying  more: drug shops in the two poorest districts had significantly higher prices. |
|  |  | All four treatments with originator brands purchased from private pharmacies were unaffordable. |
|  |  | Many participants stated that medicines are not affordable for the average person. |
|  |  | Private-for-profit (PFP) facilities are often out of financial reach for most Ugandans. |
|  |  | In practice, restricted antenatal services due to financial constraints were also reported. Men in this study perceived user fees as responsible for delayed women's care |
|  | Poor health facility access   - Poor availability of medicines in lower-level facilities - Long waiting times - Unavailability of medicines - Delay in care | Benzathine penicillin G, amoxicillin, and azithromycin, all listed on WHO EML, were less likely to be available at primary healthcare facilities, including health centres III and especially at health centres II. |
|  |  | We face serious problems managing waiting time due to increased utilisation and inadequate staff and facilities. |
|  |  | Long wait times at the health facilities.  There is some inequity in treatment between insured women and those who pay for the consultation. Insured women wait longer than others before being received by the medical staff. |
|  |  | The time will be spent waiting to buy the medicines from the pharmacy box. |
|  |  | However, access to essential medicines is not as predictable outside Maputo city. |
|  |  | The pandemic impacted the ease of essential medicine access for both acute and chronic conditions. Overall, those who had acute illnesses during the lockdown had 72.0% essential medicines accessibility, whereas those with chronic conditions had 65.0% accessibility. |
|  |  | Long waiting times at the health facility were perceived as a barrier to receiving antenatal care child vaccination services |
|  |  | Delay in receiving appropriate care. |
|  |  | The mean (median) reported delay in seeking hospital care was 3.6 (2.0) days in government hospitals and 4.4 (1.0) days in district-designated hospitals. |
|  |  | Delays in receiving appropriate care at health facilities were often owing to a shortage of skilled birth attendants. |
| Financial resources | Insufficient funding | The PHC clinic managers lamented the minimal budget and were not allowed to shift funds within the allotted budget. |
|  |  | Stock-out was due to the absence of resupply from the pharmaceutical centre and other NGOs since the beginning of the program implementation in the district. And no budget has been explicitly allocated for the program. |
|  |  | The WHO local office in Kampala and MOH Uganda continued to anticipate global level initiatives such as the Global Fund to support the rollout of child-appropriate dosage formulations - support that has not been realised to date. |
|  |  | The absence of an ENT-specific hospital budget in all hospitals in Lusaka, Eastern, North-Western, and Central provinces. |
|  |  | Insufficient spending to procure sufficient medicines for patients' needs. |
|  |  | Two clinics reported financial constraints as a possible cause of vaccine shortages. |
|  |  | A financial resource deficit for specific procurement has also been reported. |
|  |  | Poor financing by the county government. |
|  |  | The review process is long due to insufficient funds. |
|  |  | Funding allocated by the MOHW is inadequate to cover the cost of pharmaceuticals needed for the treatment of cancers diagnosed annually |
|  |  | Poor financing of supply chain management (SCM) for medicines. |
|  |  | The MoH's budget was inadequate to meet the population's needs, resulting in prioritising other diseases or health needs other than diseases related to ICEMs. |
|  |  | This study showed that insufficient funding was one of the contributors to drug stock-outs at the health facilities. |
|  |  | Underfunding of the anaesthesia service. |
|  |  | The panellists noted inadequate domestic and external funding targeted at maternal, newborn and child health problems. |
|  |  | Essential Medicines and Health supplies (EMHS) funding allocations to Uganda's public sector health facilities do not match the facilities' EMHS needs based on the number of patients they serve. |
|  |  | Only two (2.2%) schools had any fiscal allocation to school health services (SHS) from the imprest account of the headteacher's office. |
|  |  | Insufficient funds from the central government to meet local drug demands. |
|  |  | The ratio of pharmaceutical products and hospital budgets is low and different between the hospitals. |
|  |  | There is insufficient funding for the Integrated Management of Childhood Illness (IMCI) activities at the national, district, and facility levels. |
|  | Poor effect of financial autonomy | We found less evidence of the effect of the increased facility financial autonomy. |
|  | Delayed payments from insurance companies | Late claimant rebates. |
|  |  | The hospitals depend very much on funding from the NHIA to the extent that cash flow problems often reduce their purchasing power. Reducing purchasing power results in a shortage of drugs and non-drug consumables and limits initiatives. |
| Human resources | Shortage of staff   - Few specialists available - Few skilled health workers - Poor equitable access to health workforce (rural vs urban areas) - Lack of human resources - High workload | Few anaesthetic providers and a high patient load. |
|  |  | The availability of staff trained in antenatal care services and EmONC in the last two years was generally low, possibly due to the socio-political crisis in the country over the past five years. |
|  |  | The observed scarcity of human resources (trained providers contributed to the readiness of the facilities to provide BEmONC. |
|  |  | The anaesthesia workforce consisted predominantly of non-physicians, and the study also indicates low specialist level. |
|  |  | The quality of public facilities was described as poor, inconvenient and managed by unqualified healthcare providers such as trained- traditional birth attendance (trained-TBAs). |
|  |  | Shortages of nursing supply affect access to primary health care. Shortages and uneven geographical distribution of health care workers are reported. |
|  |  | Shortage of staff is one of the major ones facing the facility. This shortage in staffing presented worse dilemmas when it came to scheduling the duties for nursing staff. |
|  |  | The overburden of work made the store manager extremely exhausted and overwhelmed, and absences of indemnity for staff exacerbated the problem. |
|  |  | The nurse to patient ratio varied in different hospitals, but it was noted that there were shortages at some hospitals |
|  |  | Providers commented on the lack of human resources at health centres, with some attributing increased queues. |
|  |  | The heads of facilities complained about the insufficiency of personnel. Our study showed that none of the health facilities had a laboratory technologist. There were no laboratory technicians, no pharmacy technicians, only one of the facilities had a laboratory, and only one doctor covered the entire LG PHCs. |
|  |  | Poor distribution of health resources with a skew towards urban compared to rural areas. |
|  |  | Due to shortages of staffing at the public facilities, the population frequently turns to CHAM facilities for health care. |
|  |  | Administrative clerks or support officers assisted professional nurses with ordering medication, preparing chronic prescriptions for the pharmacy, storing medication in medicine rooms and replenishing drug stock in consultation rooms. |
|  |  | Only one (1.1%) school benefited from the services of a doctor. There was one (public secondary) school without any attending health personnel. |
|  |  | Acute shortage of staff, especially at peripheral health facilities, seemed to be the main obstacle to the effective IPTp service delivery in both study districts. |
|  |  | The person submitting the form was often the only employee at the health facility, and as the trip could stretch over a couple of days, there would be no one to care for the patients at the health facility. |
|  |  | The country has inadequate human resources and suffers from shortages of qualified health workers. |
|  |  | Facilities may hire staff specifically to take care of claims processing to ease the burden on health workers to focus on their core duties. |
|  |  | An inadequate number of clinicians and nurses in outpatient clinics at some facilities has a high workload, outpatient clinics at some facilities. |
|  |  | An unreasonable workload was indicated by a doctor working at 27 clinics alone. |
|  |  | The non-collection of routine blood was due to a high workload, a lack of trained NIM-ART nurses, and specimen bottle unavailability. |
|  |  | Key informant interview results indicated that there were low trained health workers and high turnovers of trained HEWs resulting in the interruption of the implementation. |
|  | Inadequate capacity   - Inadequate capacity of essential medicines committee - Shortage of qualified health workers - Inadequate number of health workers per facility/station | There was little to no consideration of cost-effectiveness criteria, evidenced by the committee having no knowledge of cost-effectiveness. |
|  |  | The lack of trained staff hindered the implementation the Integrated Management of Neonatal and Childhood Illness implementation (IMNCI). |
|  |  | Very few providers had received recent ANC training (16.7% at CHU, 7.0% at referral hospitals and 9.4% at CSBs). |
|  |  | The country has shortages of qualified health workers. |
|  |  | It is recommended that facilities hire staff specifically to take care of claims processing to ease the burden on health workers to focus on their core duties. |
|  |  | An inadequate number of clinicians and nurses in outpatient clinics at some facilities. |
|  |  | The non-collection of routine blood was a high workload, a lack of trained NIM-ART nurses, and specimen bottle unavailability. |
|  |  | In 4 (16%) clinics, no staff were trained in the management of TB and another three clinics, staff were trained in the management of patients with STIs. |
|  | Inadequate training   - Poor equitable training (lower clinics and health centres; rural and urban) - A low number of trained health workers | Fifty-eight percent (n=107) of nurses interviewed had been trained in Integrated Management of Neonatal and Childhood Illness implementation (IMNCI). |
|  |  | Most of the nurses reported that the Integrated Management of Neonatal and Childhood Illness implementation training was inadequate. |
|  |  | Lower clinics, health centres and medium clinics (1%, 3% and 4%, respectively) were the least likely to have trained staff compared with other facility types. The number of providers who received in-service training for diabetes is very low. |
|  |  | Rural based health workers reported that they were still using the outdated IMCI treatment charts of 2001. |
|  |  | The availability of health providers working in antenatal care who had received technical updates in ANC over the last two years was low. |
|  |  | The training of clinical staff (N=128) to deliver PHC services and treat patients with TB, HIV, or STIs was inadequate, with only 29% (37/128) and 21% (27/128) of the staff, respectively, being trained to deliver TB and ART services. |
|  |  | Key informant interview results indicated that there were low trained health workers. |
| Education system | Inadequate training | Lack of expertise was reported |
|  |  | Lack of in-country skills and capacity in supply chain management of medicines. |
|  |  | There was a shortage of nurses specialised in critical care |
|  |  | Among all facilities that offer diabetic services, only 6 % of them had at least one interviewed provider of services who received in-service training on diabetes during the 24 months preceding the survey. |
|  |  | Other barriers to quality reported by the health workers was lack of training in standard type 2 diabetes care. |
|  |  | Only one informant said he had received training about using economic evidence. |
|  |  | Training on aspects of the medicines supply chain is mainly conducted at postgraduate and degree education levels. |
|  |  | No continuing medical education is required or provided; no formal resuscitation training is provided.  Lack of educators to train the number of anaesthesia residents and technicians. |
|  |  | Few sites received any training to diagnose and manage cardiovascular diseases in the previoustwo2 years. |
| Clinical supervision | Insufficient/irregular supportive supervision | A challenge with the accreditation model has been providing regular supportive supervision to the dispensers due to time or resource limitations. |
|  |  | Low scores on supervision visits: Less than a quarter (15.5%) of the HCWs reported being supervised on NCD (HTN and diabetes) management in the last three months. |
|  |  | Difficulty providing adequate supervision of junior clinicians: low doctor- and nurse-to-patient ratios, frequent rotation of trainees. |
|  |  | Although support supervision is one of the strong components of the Pull system, it has not been effectively implemented. |
|  |  | The proportion of all health posts (HPs) that had direct case observation was only 2.4%. |
|  |  | Although a higher proportion (38%) of HSAs reported receiving a CCM-specific supervision visit in the previous three months, only 16% of HSAs reported a CCM supervision visit that included the observation of a sick child consultation |
|  |  | Within three months before the survey, less than 60% of the HSAs reported receiving any type of supervision visit. |
|  |  | The combination of stavudine and zidovudine was wrongly prescribed, and lamivudine was omitted. The clinical mentor subsequently corrected this patient's prescription. |
|  |  | They had not been oriented on the new treatment policies, and support supervision had diminished accordingly. |
|  | Lack of supervision | Lack of sustainable supervision from the Woreda Health Office, Zone Health Department, and pharmaceutical supply agency regarding on availability of tracers' drugs and the implementation of their logistic management information system worsen the situation. |
|  |  | Some physicians mentioned that there is no monitoring of their prescribing behaviour. |
|  |  | All pharmacists mentioned that they follow the proper dispensing practices, but there is no monitoring. |
|  |  | The majority of nurses were not getting any supervision when they had training. |
|  |  | The reliance of the country's PHC system on this cadre of staff with little or no supervision has placed the primary health care system in danger. |
| Internal communication | Poor coordination | Lack of stakeholders' coordination in setting short- and long-term goals to avail this essential equipment and drugs. |
|  |  | Health managers developed programmes or campaigns and neglected to liaise timeously with the provincial supply chain team, which could, in turn, result in stock-outs. |
|  |  | Also influencing care decision making were health system resource constraints such as management issues, including leadership and interpersonal relations among staff. |
|  |  | Information sharing between health centres was thought inadequate. (Seven levels of health service delivery). Referral of patients was argued to occur often, not because contact between the different levels was lacking. Lower-level centres were unaware of ICEMs availability at higher levels, hampering service delivery. |
|  |  | It was evident from the participants that the suppliers did not typically communicate with the hospitals in advance on issues that have the potential of causing medicine shortages |
|  |  | Health centre clinicians in several districts reported that they resisted supplying HSAs with drugs at first because they had not been informed about the CCM program and drug supply policies. |
|  |  | During this process, there has been recognition of inefficiencies in the system; the disconnect between the procurement list at the Medicines Stores Department (MSD) and NEMLIT and the National Health Insurance System (NHIS) benefits package. |
|  |  | Poor coordination: Expiry due to treatment policy change and duplicate procurement.  Vertical programmes quantify some medicines. |
|  | Poor communication network | Our mobile phone network is a serious challenge...Therefore, when we have an emergency case, how to link with the national ambulance or the ambulance at Nadowli hospital is always a problem. |
| External communication | Poor communication practices | Lack of information/education/communication materials and lack of job aids for provision of standard type 2 diabetes (T2DM) care. |
|  |  | Information for consumers was poor, especially when buying from drug shops. Dispensers at shops and facilities provided poor levels of basic information to buyers. |
| Allocation of authority | Lack of health workers' involvement | The pharmacists and the technicians acclaimed that the hospital managers exclude them from the important meeting of the hospital where the decisions tackling medicines, drug supply and procurement are taken. |
|  |  | Non-participation of clinicians in medicine quantification in hospitals: no advice from expert clinicians on medicines forecast. |
|  | Limited authority   - Lack of autonomy - Poor decentralization of authority | The community members nor the facility managers have direct control over the community health fund to utilise the money. |
|  |  | Tanzania uses an integrated logistic system. With this system, the facility places its order through the District Medical Officers' office to the Medical Department Store. Hence, any faults or delays in the ordering system may result in the facilities' low availability of items or services. |
|  |  | The PHC clinic managers felt they lacked control over the facility budget, staff, and supply chain management. |
|  |  | Community-based health insurance (CBHI) financing is handled by those least concerned with health and unable to produce the bill on time for the purchase/refill of supplies. |
|  |  | Tanzania also does not have an agency devoted to controlling medicine prices. |
|  |  | Health managers developed programmes or campaigns and neglected to liaise timeously with the provincial supply chain team, which could, in turn, result in stock-outs |
|  |  | The MOHW budget may be subject to delays in releasing funds, which leads to delays in disbursement. |
|  |  | One of the participants suggested that the Central Medicines Depot should take over the buy-out of medicines listed on the EML on behalf of the hospitals to ease the burden of this process. |
|  |  | Lack of clarity over human resources of health management roles at the county level. |
|  |  | In some cases, supply chain design and decision-making structures that are technically optimal lead to conflict between district and federal level health officials over which party is truly responsible for health system performance. |
|  |  | Decentralize the implementation and oversight responsibility to the district level. |
|  | Disconnected health committees | Currently, there are two parallel committees for EML and STGS; hence may lead to policy duplication or omissions, eventually negatively affecting access to medicines and therapeutic outcomes. |
| Accountability | Poor Tracking/Management system | All APTS and 10% of non-APTS hospitals were able to conduct a transaction audit at the end of 2018. APTS hospitals had a daily sales tracking/management system, but none controlled hospitals. |
|  |  | Some drugs like mebendazole were unaccounted for in entire the stock. |
|  | Inadequate of accountability   - Inconsistent records - Lack of transparency | The present study showed a 100% discrepancy between recorded balance and physical inventory, while the mean discrepancy was 60.4%. |
|  |  | Transparency over the utilization of drug funds, both from the central government and those locally mobilized through the community health fund (CHF) scheme, was very low. |
|  |  | Kenya Essential Medical Supplies Authority (KEMSA) have not been distributing these cancer drugs to these health facilities resulting in a high stock-out rate of anticancer medicines. |
|  |  | The MOHW budget may be subject to delays in releasing funds, which leads to delays in disbursement. |
|  |  | Government failure to formally adopt child-appropriate dosage formulation Because dispersible tablets were perceived to be more expensive, the MOH was reluctant to integrate some child-appropriate dosage formulations into the EMHSLU 2012 and scale up their distribution countrywide. |
|  |  | An analysis of the clinical errors made by HSAs indicates that substantial proportions of children presenting with fever and diarrhoea may have received inappropriate treatment due to stock-outs of antimalarials and ORS, respectively. |
|  |  | The lack of equipment in district referral hospitals may be due to the weak support from the ministry of health to this lower level of public hospitals. |
|  |  | Community-based health insurance financing is handled by those least concerned with health and unable to produce the bill on time for the purchase/refill of supplies. |
|  |  | Given comparatively poor program results for paediatric HIV/AIDS, medication shortages for children require special attention. |
| Management and/or leadership | Inadequate capacity | The Ministry of Health should strengthen its leadership and coordinate the support and collaboration of partners and stakeholders to work on the public health facility's capacity to deliver services. |
|  |  | To improve BEmONC services, health system leaders should implement strategies to ensure better fair distribution of clinical guidelines, essential medicines, equipment, and refresher training. |
|  |  | Lack of stakeholders' coordination in setting short- and long-term goals to avail this essential equipment and drugs. |
|  |  | The Health Facility Governing Committee (HFGC) members do not have strong financial management skills such as budgeting, clear record accounting, monitoring, and reporting. |
|  |  | A lack of trained nurses prevented the effective implementation of the NIM-ART programme. |
|  | Inadequate support | Rural based health workers reported that they were still using the outdated IMCI treatment charts of 2001. They had not been oriented on the new treatment policies, and support supervision had diminished accordingly. |
|  | Lack of knowledge and skills | Nurse ward managers lacked specific training in quality improvement, and they were also worried about the knowledge deficit in this critical area of their managerial role. |
|  | Lack of leadership | Also influencing care decision making were health system resource constraints such as management issues, including leadership and interpersonal relations among staff. |
| Information systems | Inadequate capacity   - Poor ICT skills and practices | All sites relied on paper-based longitudinal records, and relatively few had a functional computer or access to the internet or email. |
|  |  | They complained that the pharmacy staff did not use the software (RxSolution) appropriately, making inventory management a difficult task. |
|  |  | National information systems are too weak to accurately generate and analyse information on health status and determinants of health of women, newborns and children. |
|  | Inadequate information system   - Lack of reporting procedures - Lack of structured ordering systems/methods - Lack of stock control mechanisms | Experience challenges in logistics management information system |
|  |  | Nigeria does not have a specific reporting procedure for compounded products, even though a general pharmacovigilance reporting system has been operating since 2004. |
|  |  | Uganda has no system to document the use and need of internationally controlled essential medicines (ICEMs). Instead, quantification is based on estimates, leading to inadequate quantities and stock-outs |
|  |  | Lack of use of standard ordering methods |
|  |  | 64% (16/25) of the facilities had no stock control mechanisms for essential drugs and supplies. |
|  | Poor information management practices   - Poor record-keeping - Lack of logistics and management system - Lack of standardized treatment protocols - Poor monitoring of inventory levels | Poor information system is reported as a challenge. |
|  |  | Some hospitals did not have proper records to calculate stock-out days of medicines for at least the past six months. |
|  |  | The lack of standardized treatment protocols limits the ability to predict prescribing patterns and forecast the volumetric quantity of drugs needed. |
|  |  | The study found that six out of the nine health facilities did not have computerized Logistics and management Information systems (LMIS) in the store.  The current study revealed that store managers did not fill bin cards and stock cards, which compromised the quality of the report and requisition form (RRF) sent to pharmaceuticals funds and supply agency (PFSA). |
|  |  | However, some records on ICU admission cases were missing in Private and Mission hospitals. |
|  |  | Although there is a guideline to use LMIS in this HC, the absence of a computer software system and lack of training on logistic forms result in poor record keeping practice. |
|  |  | Weak logistical information systems were also noted in the study. |
|  |  | A total of 59 (64.8%) schools kept no records of school health services at all. Of those who did, 23 (25.3%) kept noncumulative records, while only 8 (8.8%) schools had cumulative and transferrable records. |
|  |  | Inventory levels are not regularly monitored, and they lack knowledge of basic expiry prevention tools. |
|  | Use traditional manual systems | Excessive documentation: hospitals still rely heavily on manual methods for processing claims. This traditional manual method causes delays in processing claims. |
|  | Poor availability of reports | Not all reports for visits affected were available since, in some cases, findings from the monitoring visits were communicated verbally with no documented reports made. |
|  |  | The need for specific medications is highly variable and forecasting medication supply needs were initially a challenge in the absence of appropriate data. |
|  | Lack of registries | Lack of standardized RHD case management systems and the absence of clinical case registries, the magnitude of the RHD problem is underestimated in their districts. Hence, administrators do not prioritize RHD in their budgets. |
|  |  | Currently, Ethiopia has only one cancer registry canter established in a central referral hospital. |
|  |  | The patient-retained card indicates a return date in all clinics, but this is not captured in the clinic in an appointment register. |
|  |  | All clinics had a chronic disease register but no diabetes-specific structured care form. |
| Facilities | Poorly resourced health facilities | Many facilities have not been upgraded to provide EmONC and neonatal care as intended through the KEPH. |
|  |  | Most participants thought they were not equipped with the necessary resources by the national or relevant provincial health department. |
|  |  | Procedural sedation facilities and medicines were generally good in the private sector but poor in the public sector. The difference was attributed to a lack of equipment, staff and protocols in public-sector facilities. |
|  |  | The medical centre was perceived to have a limited treatment capacity and would refer patients to higher levels of care in case of complications. |
|  |  | Although most PHUs had the necessary facilities and equipment for providing maternal and neonatal health care, these were often not in satisfactory condition. |
|  |  | The proportion of obstetric doctors per 10 000 births at the 15 hospitals did not correlate significantly with the maternal mortality ratios. |
|  |  | The non-collection of routine blood was due to a high workload, a lack of trained NIM-ART nurses, and a specimen bottle's unavailability. |
|  | Sub-optimal availability of medicines   - Poor availability of medicines in lower-level facilities - Pandemics impact the availability of medicines - Facility level stock-outs - Poor equitable availability of medicines in lower-level facilities | Injectable insulin was expected at HC-IVs and hospitals but was only observed in 50% (17) of these facilities and 11% (22) of all facilities. |
|  |  | The pandemic impacted the ease of essential medicine access for both acute and chronic conditions. Overall, those who had acute illnesses during the lockdown had 72.0% essential medicines accessibility, whereas those with chronic conditions had 65.0%accessibility. |
|  |  | Most pharmacists (69.5%) reported experiencing stock out of essential medicines in their facility. |
|  |  | Sixty-eight percent of medications considered first, the second, and third-line to treat these conditions were absent from the hospital formulary. |
|  |  | There were wide variations in EMHS allocations per patient within the same levels of care. HC IVs and regional referral hospitals had the widest disparities in patient load and, therefore, in allocation per patient. |
|  |  | HC IIs reported the lowest (63 %) average availability of vital EMHS on the day of visit and hospitals the highest, with 88% for general hospitals and 87% for regional referral hospitals. |
|  |  | General hospitals had the shortest average time out-of-stock at 8 %, and HC IVs had the longest average time out-of-stock (28 %) |
|  |  | A greater percentage of facilities that placed their orders had optimal stock levels, while lower-level facilities that received kits were predominantly under or overstocked. |
|  |  | Among 230 EDs checked for availability based on the list of drugs for health centres, only 128 (55.65 %) drugs were available. |
|  |  | The availability of medicines in public and faith-based sectors was often much lower than the 80% availability target set by WHO for NCD medicines. |
|  |  | Good policy is not enough to sustain reductions in child mortality; reliable supply and access to effective essential drugs are critical. |
|  |  | The availability of key NCD medicines for the management of diabetes and hypertension is suboptimal. |
|  | Low availability   - Lower availability of medicine in public facilities - Lower availability of medicine in lower-level facilities - Low availability of NCDs medicines - Low availability of obstetric medicines - Blood and blood products insufficiency - Shortage of antibiotics - Low availability of oxygen | There were consistent patterns of lower availability in government facilities than mission/ faith-based and private facilities, with lower availability in dispensaries and health centres than in hospitals and lower availability in rural than urban health facilities. |
|  |  | Low availability was noted for these medicine categories in public hospitals compared to private hospitals and pharmacies. |
|  |  | The availability of basic medicines required for the treatment of diabetes in Ethiopia is very low.  The availability of medicines for diabetes varies by facility type and is higher in hospitals when compared with other facility types (ranging from 90 % in hospitals to 0 % in clinics). |
|  |  | The availability of the thirteen key life-saving commodities to prevent and treat major causes of maternal, newborn, and child death is generally low, except for amoxicillin at primary care facilities and first referral hospitals. |
|  |  | The availability of essential medicines for delivery, intravenous fluids (48.15%), injectable magnesium sulfate (40.77%), and injectable antibiotic (32.11%), were not widely available. |
|  |  | The availability of emergency drugs and essential equipment was generally inadequate. |
|  |  | Low availability of Cardiovascular drugs. Half of diabetes and asthma drugs were available in the health centres. Some NCD drugs were not available at all during the study. |
|  |  | Low availability of essential medicines in the public sector facility. |
|  |  | There is a shortage of drug supply. |
|  |  | The drug availability in obstetric care is very low. |
|  |  | The availability of BEmONC tracer items at the primary care level was very low. The CSBs had only about five of the thirteen (38%) tracer items available on site. Blood sufficiency, defined as no interruption in blood availability in the past three months, was reported at half university hospitals and 44.4% of regional referral hospitals. |
|  |  | Only 0.1% of medical centres and 0% of peripheral health centres had the whole set of essential medicines tracer items. Essential medicine availability of less than 1% for both levels. |
|  |  | Eleven items were out of stock in 9 of 31 clinics on the visit. |
|  |  | There is a low supply of endogenous essential drugs supply shortage. |
|  |  | Lack of essential medicines and equipment for nurses to use in the  Ward. |
|  |  | Mean availability across all surveyed antidiabetes and anti-hypertensive medicines in the surveyed pharmacies was low (30-<50%). |
|  |  | In this study of public-sector health facilities and a census of district-level drug warehouses, we found that essential medicines for mental healthcare were routinely unavailable. |
|  |  | Results of the survey depicted that among all facilities offering diabetes services, only 11 % of them had metformin, 28 % had Glibenclamide, 9% had injectable insulin, and only 15 % had injectable glucose solution on the day of the visit. |
|  |  | Eighty-five percent of the 13 district depositories had a stock-out of an essential drug. |
|  |  | There is frequent stock out of essential drugs. Essential medicines are available in only 50% of lower-level health facilities (dispensaries and health centres) and about 65% of hospitals in Kenya. |
|  |  | Shortage of essential drugs, especially for treating pneumonia, diarrhoea (ORS) and malaria, was also identified as a major challenge in IMCI implementation. |
|  |  | Shortage of antibiotics, stock-out of penicillin, and antidiabetic medicine were reported in the urban unit. |
|  |  | Also influencing care decision making were health system resource constraints such as staff availability, medicines, and supplies such as oxygen and equipment. |
|  |  | We found significantly lower availability of NCD medicines listed in the list than medicines for acute conditions. |
|  |  | None of the dispensaries could administer one or all essential drugs: anti-convulsant, uterotonics or antibiotics. None of the dispensaries had a complete birth kit. |
|  |  | Low possession of essential drugs and medical disposables in many selected healthcare facilities. |
|  |  | ICEMs were also not always available in the public sector, where all medicines are free to the patient, forcing patients to seek care from the private sector, where costs are substantial. |
|  |  | Shortage of blood and blood products for transfusion. Shortages in regional anaesthetics supplies throughout the country and other essential drugs like antacids and anti-hypertensive drugs |
|  |  | The availability of medicines to treat chronic diseases in children was very low. |
|  |  | All health facilities surveyed reported having adequate delivery packs. However, there were shortages of some essential drugs and supplies needed for EmOC in most facilities on the day of the survey; oxytocin and ergometrine. |
|  |  | Purchases of medicines or medical supplies were due to shortages in the hospital or ward and included essential items such as quinine, paracetamol tablets and syringes. Sixty-six per cent of caretakers purchased the medicine at private pharmacies, and 33% from the hospital pharmacy. |
|  | Unavailability of medicines   - Stock-outs of essential medicines for NCDs - Lack of paediatric formulations - Unavailability of essential medicines in public facilities - Lack of analgesics drugs | Stock-out for essential medicines for non-communicable diseases. All the cancer medicines were unavailable except for methotrexate and morphine solution for palliative care available in only one of the five hospitals visited. |
|  |  | All facilities surveyed reported experiencing stock-outs of at least one class of essential medicine for NCDs within the previous year. |
|  |  | The availability of essential drugs was generally low, especially nifedipine and metformin, lower than 80% of the WHO availability target for NCD management in health facilities. |
|  |  | The mean availability of surveyed CVD essential medicines was lower than that of the WHO Core EMs in private and public-sector pharmacies. |
|  |  | All antimalarial drugs, including oral continuation therapies, experienced stock out during the last three months before the study. |
|  |  | At least 13 of the 33 drugs on the Botswana NEML were out of stock for defined periods ranging from 10 days to 211 days. |
|  |  | Paediatric formulations of amoxicillin are not available in Nigeria. |
|  |  | The lack of essential drugs and supplies hindered the implementation of IMNCI |
|  |  | Poor income groups used only the public sector for all healthcare services. Still, they were sometimes forced to use private-sector pharmacies due to shortages or unavailability of medicines at the public sector clinics. |
|  |  | Most participants stated that medicines were not available at the government facilities, which forced them to buy the medication at private facilities and pharmacies. |
|  |  | Of the 48 facilities studied 10 had no essential drugs for HIV, Malaria and TB even though facilities provided services to manage such paints. |
|  |  | The average stock out days of tracer's drugs (TDs) from all five HCs within the past 12 months was 40.6 days. Among five HCs, ORS was stock out for a more extended period (144 days), whereas paracetamol was stock out for a shorter duration of 1.4 days. |
|  |  | Essential drugs for Spinal Anaesthesia and General Anaesthesia were not always available. Analgesics were poorly available except for oral paracetamol and pethidine. |
|  |  | Delay in receiving appropriate care due to lack of essential drugs and supplies was cited as a hindrance to health facility delivery. |
|  |  | Essential second-line drugs, such as tranexamic acid, prostaglandin F2-alpha, and ergometrine, were unavailable in several institutions. |
|  |  | The participants highlighted stock-outs at MSD as the major problem of the drug supply chain. malaria, essential medicine went out of stock faster than expected at the health facilities |
|  |  | 80% (20/25) of facilities reported non-availability of key drugs and supplies six months before the audit, and 64% (16/25) had no stock control mechanisms for essential drugs and supplies. |
| Patient flow processes | Inadequate referral system | Several instances were cited where clients refused referrals for logistic and service responsiveness reasons such as the travel distance, time and money involved, and the uncertainty of the kind of reception they would get at the receiving hospital. |
|  |  | Inadequate referral service (81.7%) was the factor that affected the utilization of university health services the most. |
|  |  | Referral of patients was argued to occur often, not because contact between the different levels was thought to be lacking. Lower-level centres were unaware of ICEMs availability at higher levels, hampering service delivery. |
|  | Poor referral practices | Most of the patients in this study were referred to hospitals without receiving pre-referral antimalarial treatment, and many of them spent a long-time receiving care. |
|  |  | In some cases, patients travel without referral letters and, as a result, default on their treatment because they are apprehensive about going to the health centre without a referral letter. |
|  |  | Good referral practice was found in only 11.1% of referred patient |
|  |  | The professional staff shortages at Nadowli District Hospital were found to be contributing to staff role stress and unnecessary referrals of pregnancy and newborn cases to other hospitals. |
|  |  | Dhaka and Matlab Hospitals did not maintain antiretrovirals because patients with suspected or confirmed HIV infection are referred to government facilities. |
| Procurement and distribution systems | Poor training in procurement | Lack of training in the system of procurement.  Due to a lack of training, facilities do not always promptly make medicine and commodity requests, preventing facilities from offering the services and medicines required by the KEPH. |
|  | Poor procurement funding   - Limited funding | Overall, access to microfinance did not influence the stock availability of the drugs. |
|  |  | Delay in reimbursement significantly reduces our capacity to pay suppliers, purchase drugs and carry on effective administrative work. |
|  |  | With limited budgets, procurement of essential medicines was further prioritised using the VEN (vital, essential, necessary) classification. |
|  |  | Whereas some child-appropriate dosage formulations were available globally, poor countries like Uganda could not easily access them because they were initially too expensive. |
|  |  | Cost, budget, tenders, and price negotiation affect medicine selection. |
|  | Distribution monopoly | The drug distribution monopoly of the Medical Stores Department (MSD) to public health facilities and the transfer of the yearly budget for drugs and medical equipment directly from the government to the MSD left no alternative option for purchasing drugs or medical items when MSD was out of stock. |
|  | Inappropriate selection | We are phasing out medications without the involvement of health workers (HWs), contributing to discrepancies in order placements. |
|  | Inefficient distribution systems   - Ineffective distribution of supplies at district levels - Delay in servicing orders - Lack of transport - Long delays in supply - Supply of expired and unneeded medical supplies | Most facility-level drug and supply stock ruptures resulted from central or widespread stock-outs and failure to effectively distribute stocks at the district warehouse. |
|  |  | Some participants stated that PFSA could not be able to supply TDs, such as Ferrous Sulfate with Folic Acid and Tetracycline eye ointment, even if they requested a timely.  Transportation problem was also mentioned as a reason for stock-outs of TDs. |
|  |  | Providers in rural CHCs highlighted the lack of dedicated vehicles to transport medicines from CHCs that act as temporary storage for rural clinics as a hindrance to efficient distribution. |
|  |  | Stock-outs resulted from the following inefficiencies at the central level: community-based medicines distribution systems, which deliver the CDU‚ prepacked medicines to non-health facilities nearer to patient homes. |
|  |  | There is maldistribution of essential drugs despite stock out of public facilities. |
|  |  | Participants reported the long delays and the supply of expired drugs and unneeded medical supplies, suggesting the distribution system's ineffective functioning. |
|  |  | Orders take long before they are supplied from the depot, due to lack of dedicated van for each facility affecting pick up of orders. |
|  |  | The distribution system was also thought to be a barrier. Health centres only receive a fraction of the ICEMS orders due to problems experienced by the distributor or due to practical delivery issues. |
|  | Inefficient procurement processes   - Delay in servicing orders - Under-supply and over-supply of medicines - Unreliable supplies - Logistical challenges - Long procurement processes - Poor servicing of medicine orders - Unreliable logistic management | The median number of days out of stock for drugs and medical supplies was higher in the Pull system than in the Push system. |
|  |  | Monthly drug stock deliveries were lower than health facility requests, with isolated months where drugs were oversupplied, followed by lower health facility requests, particularly for metformin. |
|  |  | Unreliable supplies of test kits and essential drugs and equipment led to uneven implementation of integrated HIV testing and counselling. |
|  |  | The average stock-out days were 80. This was the shortage of resupplies and refills of drugs and medicines from different non-government organizations and the district health office. |
|  |  | Under Kenya's drug supply system, health centres receive standard kits containing essential drugs from the Kenya Medical Supplies Agency (KEMSA), which sources unable to cope with health facility's needs. |
|  |  | Shortage of supplies and essential drugs, especially for treating pneumonia, diarrhoea and malaria, was also identified as a major challenge in IMCI implementation. |
|  |  | All the facility managers informed that drugs and medical materials were supplied monthly and that these supplies were enough for only the first two weeks of the month. |
|  |  | Shortage of blood and blood products for transfusion.  Shortages in regional anaesthetics supplies throughout the country and other essential drugs like antacids and anti-hypertensive drugs |
|  |  | Lack of essential drugs and supplies was cited as a hindrance to health facility delivery. |
|  |  | Of concern was the erratic drug supply, especially the regular stock out of essential ARV drugs at the facilities, during the study. In particular, tenofovir was regularly unavailable, and NIM-ART nurses were compelled to change patients to an alternative ARV drug regimen. |
|  |  | All health facilities surveyed reported having adequate delivery packs. However, there were shortages of some essential drugs and supplies needed for EmOC in most facilities on the day of the survey; oxytocin and ergometrine. |
|  |  | Our exploratory analysis showed that drugs that experienced a stock out had higher MPR ratios, suggesting that inefficient procurement leads to a higher likelihood of stock out or vice versa; drugs that experience a stock-out are more likely to have inefficient procurement processing as a stopgap measure. |
|  |  | All health facilities had logistical problems and insufficient supplies of medical equipment and drugs to care for patients. |
|  |  | Eleven (69%) RRHs, 15 GHs (58%), and 9 HCIVs (64%) reported they routinely did not receive the drugs requested from National Medical Stores (NMS). |
|  |  | Ten (77%) RRHs, 17 (65%) GHs, and 10 HCIVs (71%) reported they routinely did not receive the requested quantity of particular drugs. |
|  |  | According to the participants, the buy-out process is currently very lengthy. Due to its complex nature, it does not ensure medicines availability at the time of need, thus contributing to the unavailability of medicines in their hospitals. |
|  |  | Tanzania is further impacted by the lengthy ordering and delivery of drugs and medical supplies through an integrated logistic system. Hence, any faults or delays in the ordering system may result in the facilities' low availability of items or services. |
|  |  | Many participants perceived the health facilities as lacking essential resources, including medicines, childhood vaccines and logistics such as refrigerators to maintain the cold chain. |
|  |  | There is no system whereby hospitals can procure medicines not available in suitable dosage form from manufacturers. |
|  |  | Unreliable logistics management has affected the delivery of health care in Burkina Faso. |
|  |  | Long procurement processes involved in medicine purchase "Kenya Essential Medical Supplies Authority (KEMSA) have not been distributing these cancer drugs to these health facilities resulting into high stock out rate of anticancer medicines." |
|  |  | The government cites lengthy procurement processes in the health centre drug supply. Although accurate data measuring the frequency and severity of drug stock-outs is limited, it is a large health system problem in Malawi. |
|  | Poor quantification   - Poor consumption forecast at the facility level - Poor quantification at the facility level - Poor quantification for facility receiving kits | Low availability of overused essential medicine (Ems) usually occurs due to poor consumption forecasting and procurement. |
|  |  | Poor forecasting at the level of health facilities was sometimes regarded as a challenge, particularly during times of policy changes or strategic service delivery decisions that led to increased demand for specific medicines and medical devices without the necessary adjustments to the quantification and ordering processes. |
|  |  | Compliance of health system staff is key in managing stock out. Stock out is caused by poor quantification of medicine. |
|  |  | Overall, 43 % of all the facilities had optimal stock levels, while 27 % were understocked and 30 % were overstocked. |
|  |  | A greater percentage of facilities that placed their orders had optimal stock levels, while lower-level facilities that received kits were predominantly under or overstocked. |
|  | Poor stock management   - Poor tracking system - Poor logistics and supply chain management SCM) practices - Expiry of products - Inaccurate stock records - Lack of stock control mechanisms | The findings in the study point to a need for supply chain management systems that track medicine availability and cost in real time. |
|  |  | Vaccine shortages are attributed to delays in delivery, depot being out of stock, orders reduced by the district depot and shortage of vehicles to collect vaccines. |
|  |  | 16% of participants admitted having a set of SOPs for medicines logistics. (39%) could handle both local and international logistics for medicines, while 61% only handle local logistics for medicines. Pharmacists' views suggest poor logistics and SCM practices. |
|  |  | Wastage due to the expiry of products |
|  |  | Accurate stock record balance ranges from 44.4 to 100%, while the percentage of stock record balance that is less than physical inventory ranges from 0 to 33.3%. In contrast, the percentage of stock record balances greater than physical inventory ranges from 0 to 44.4%. |
|  |  | Antibiotics were also supplied to and from unapproved medicine outlets |
|  |  | 64% (16/25) of the facilities had no stock control mechanisms for essential drugs and supplies. |
|  |  | Lack of knowledge of basic expiry prevention tools. |
|  |  | The long delays and the supply of expired drugs and unneeded medical supplies were reported by participants |
|  |  | Pharmacists’ experiences challenges in supply chain management. |
|  | Poor monitoring and evaluation | Pharmacists’ experiences challenges in monitoring and evaluation. |
| Incentives | Low pharmaceutical company incentives | Low incentive for pharmaceutical companies to manufacture and sell the drug due to poor profit margins. |
|  |  | No priority is given to essential medicines with regard to encouraging local manufacturing by providing incentives such as tax incentives and subsidies. |
|  | Low motivation of health workers   - High workload - Non-payment of stipends - Lack of transport provision - Poor remuneration | Enrolled nurses who were assigned the responsibility of the management of pharmaceuticals stated their resentment and frustration with this responsibility. |
|  |  | A lack of incentives and non-payment of lay counsellors' stipends emerged as a challenge |
|  |  | Most health facilities did not provide transport (e.g., motorbikes) for midwives to visit communities, thereby restricting their ability to engage in health education to follow up on women not attending ANC. |
|  |  | The government is paying little to these health workers. Nurse managers demonstrated they had less influence on policy and resource development to ensure incentives and timely payment for nurses to improve their motivation to work and reduce absenteeism. |
|  |  | Delays in the payment of claimant rebates to health staff and the demotivation of health professionals. |
|  |  | We are having more sites accredited for ART or use non-accredited sites. As medicine collection sites and incentivizing doctors to work in rural sites so that staffing levels can be improved. |
|  | Non-payment of suppliers | Some participants stated that non-payment of suppliers is a contributing factor to medicine shortages in their hospitals. |
|  | Inadequate seller's incentives | Profit-and-incentive-biased quantification: seller's incentives influence quantification. |
|  | Poor impact of incentive programs | We did not find evidence of Pay4Performance increasing motivation. |
|  |  | Estimates suggest that the PBF intervention did not affect stock-outs of antenatal care drugs, vaccines, IMCI drugs, and labour and delivery drugs. |
|  |  | The effect of payment based on performance (group T1) versus payment not tied to performance (control group C1) and found no difference in the stock-out rate of essential medicines |
| Bureaucracy | Unsupportive bureaucracy   - National level decision making - Bureaucratic processes | Decisions are taken by the leadership at the national level and are made in a bureaucratic fashion, with little or no evidence to underpin them. |
|  |  | Community-Based Health Insurance financing is handled by those least concerned with health and unable to produce the bill on time for the purchase/refill of supplies. |
|  |  | The Ugandan MOH did not use the normative documents from WHO and their partners to initiate a medicine policy change dialogue. This, coupled with a rather hard-headed economic decision favouring lower-priced adult dosage forms, paralysed both the confidence and authority to initiate policy favouring child-appropriate dosage formulations. |
|  |  | Procurement of drugs for HCs is highly bureaucratic.  The government allow the purchase of drugs from private supplies only when unavailable in PFSA. It also limits the quantity of medication to be purchased. |
|  |  | The bureaucratic process of accessing locally mobilized drug funds: the Community Health Fund is meant to act as a complementary source of drug funds for a facility during a drug shortage. The accessibility of funds was difficult due to its complicated approval process. |
| Relationship with norms and standards | The disconnect between National EML and STGs | Lack of coherence between the Namibia essential medicine list (NEMLIST) and STG, with some (three) of the antibiotics listed in NEMLIST, having no indications in STG, and two of AB in STG not listed in the NEMLIST can lead to misuse of antibiotics. |
|  |  | Contradictions between the essential medicines list (EML) and the standard treatment guidelines (STGs) in Kenya might have inhibited the uptake of Novartis Access and contributed to the low availability of non-communicable disease medicines included in the programme portfolio overall. |
|  |  | The Kenya National EML states that most non-communicable disease medicines should only be available at subdistrict hospitals and higher levels. At the same time, local STGs do not restrict the management of common non-communicable diseases to specific levels of care. |
|  | Lack of EML | None of the facilities surveyed operated a drug revolving fund scheme nor had an essential drugs list. |
|  | Incompliant to EML | However, in the urban unit, all pharmacists (50%-60%) prescribe drugs from outside the list as there is a shortage of many drugs. |
|  |  | Less than 10% of the available FDCs in this study were included on the Nigerian or WHO model essential medicine lists. |
|  |  | Of the 3130 registered products, just over a quarter (28%; 880/3130) were listed on the EML. |
|  |  | Twelve of the NCD medicines in this study were not on the EML. However, each of these medicines was found at all levels of care. |
|  |  | 81.7% of medicines were prescribed from the Essential Medicines and Health Supplies List of Uganda (EMHSLU), slightly lower than the WHO recommended value of 100%. |
|  |  | Non-compliance with the MEML. Medicines that were supposed to be found at the district level and above found their way to the primary health centres, with 48% at this level and 68% and 82% at the district and central hospital levels, respectively. |
|  | Conflicting guidelines | The new malaria case management guidelines were identified as conflicting information with IMCI guidelines. |
|  |  | The provinces, in some cases, use different treatment protocols, and this confuses not only the patients. |
|  | Inadequate guidelines | Furthermore, while health workers reported having the basic Uganda clinical guidelines that some commended for guiding diagnosis and treatment, others indicated that the guidelines were shallow. |
|  | Inadequate access to guidelines | No national guidelines in BEmONC were available at this level. |
|  |  | The management of a failed spinal is difficult since there are no apparent protocols available |
|  |  | There are no guidelines or details for the inclusion or exclusion of medicine, a decision that is still left at the reviewer's discretion. |
|  |  | The lack of standardized treatment protocols limits the ability to predict prescribing patterns and forecast the volumetric quantity of drugs needed. |
|  |  | The inexperience of the experts involved in the review process in using an evidence-based approach, the lack of guidelines on how to do the review and time constraints. |
|  |  | The lack of local protocols and guidelines for patient management. |
|  |  | Guidelines for postoperative analgesia were only available in 5 hospitals (12%). |
|  |  | Across all basic health facilities surveyed, no staff had been trained in EmONC in the preceding two years. Similarly, no national guidelines in BEmONC were available at this level. |
|  |  | It also highlighted the gaps in the availability of important supportive items such as refresher training and clinical guidelines for BEmONC. |
|  |  | Written procedural sedation protocols were available in 70.6% of the ECs. |
|  |  | 12 % of the facilities had guidelines for diagnosis and management of diabetes at the service delivery site during the survey |
|  |  | Nearly half (48%) of HFs had a copy of 2013 national malaria treatment guidelines or other MoH reference material with malaria treatment content, for example, a primary care handbook. |
|  |  | Guidelines for postoperative analgesia were only available in 5 hospitals (12%). |
|  |  | Only 14.6% of the respondents stated that they possess or have come across a copy of the National Drug Distribution Guidelines. |
|  |  | Poor dissemination of standard treatment guidelines. |
|  |  | Low availability of guidelines for ANC and BEmONC services at the health facilities. |
|  |  | Among the facilities that offer services for diabetes, 12% of them had guidelines for diagnosis and management of diabetes at the service site during the survey |
|  |  | No guidelines for risk stratification of hypertension were available in the consultation rooms, nor were manuals or algorithms. |
|  |  | Only 4 (30.8%) RRHs, 4 (15.4%) GHs, and 1 (7.1%) HCIVs reported having access to diabetes management guidelines. |
|  |  | Fewer facilities reported having access to guidelines on managing hypertension, hyperlipidaemia, and asthma and screening and treating tobacco use, cancer, mental health, or sickle cell disease. |
|  | Incompliant to guidelines | Current antibiotic indications are not in tandem with the Namibian National Institute of Pathology resistance reports, which may lead to a loss of sensitivity to essential antibiotics such as cotrimoxazole, azithromycin and amoxicillin (CAA). |
|  |  | Assessments demonstrated that the organisation of primary eye care does not meet expected standards as specified in the National Department of Health PHC guidelines. |
|  |  | None of the dispensaries complied with the guidelines to provide basic EmONC. |
|  |  | Over 40% of non-bacteria diarrhoea in children received antibiotics, not in line with the STG. |
|  |  | The two most available medicines were oral salbutamol tablets and oral corticosteroids neither on the WHO EML nor part of the guideline-recommended mainstay treatments. |
|  |  | MoH officials admitted that it has not been possible to adapt new WHO IMCI algorithms. |
|  |  | Poor adherence to standard treatment guidelines. |
|  |  | Non-adherence to laid down protocols in delivering healthcare |
|  |  | The clinical notes review demonstrated that adherence to recommended care was inconsistent* |
|  |  | Only 2.2% of the patients were appropriately treated according to the guidelines |
|  |  | Standard treatment guidelines are violated in forecasts. |
|  | Practice incompliance   - Improper practice - The disconnect between policy and practice - Irrational use of drugs | The study shows that obstetric care providers did not practice properly |
|  |  | There were contradictions between policy and practice that affected the adoption of child-appropriate dosage formulations. |
|  |  | Of the 566 essential medicines and vaccines, nearly half (49%; 275/566) had no registered product in 2012. |
|  |  | While most of the fixed-dose combinations (FDCs) were registered with the Nigerian drug regulatory agency, some of the varieties were found to be irrational. |
|  |  | A dangerous market norm had developed of selling part-doses. 15% (26% in drug shops) of exit interviewees had purchased only a part of the dose. Interviews showed that dispensing of part doses was a norm in private sector dispensing and was highly prevalent in NGO facilities. |
|  |  | In the University Hospital Centres (CHU), the non-conformed prescriptions are represented by 53% of the prescriptions. |
|  | Lack of standard operating procedures | None of the clinics had written standard operating procedures (SOPs) for drug receiving, storing and dispensing. |
| **Theme 5: Level – Social and Political constraints** | | |
| Ideology | Community beliefs and attitudes | Beliefs and attitudes of the community and patients negatively influenced the use of ICEMs. Communities associate ICEMs with diseases in bad faith and patients who are known to be taking medicines for such diseases are socially excluded and isolated, such as is the case for epilepsy. |
|  |  | Patients fear addiction, and they associate some ICEMs with death because patients with end-stage diseases receive palliative care, which sometimes includes opioids, to alleviate their pain. |
|  | Market ideologies | It was found that the process for a generic entering the market was quite simple as it might be added onto a formulary without a review process if all preliminary criteria were met. |
|  | Political ideologies | The distribution of these facilities in areas with no or few inhabitants lends credit to the belief that most of these centres are built for political and economic purposes and not with the involvement of the communities. |
|  | Prescription contradiction   - Insurance and prescribers disconnect | National Health Insurance Drug lists for Community-Based Health Planning and Services (CHPS) compounds prohibit the prescription of antibiotics to newly delivered mothers, despite it being mandatory. When we prescribe it, NHIS refuse to pay. |
| Contracts | Absence of structured contracts | Participants pointed out that tenders were sometimes given to companies that could not deliver, which has important implications for the patient care within their hospitals. |
|  |  | Absence of national contracts for medicines on the provincial code list |
|  | Tender delays | Delay in award of pharmaceutical tenders. |
| Legislation or regulations | Abrupt changes in policies | The key informants felt that abrupt changes in policies might also cause the expiry of drugs. |
|  | Unclear policy adoption | Medicine policies in Namibia currently promote wide use of cotrimoxazole, amoxicillin and azithromycin (CAA) antibiotics by the level of care and disease, which may lead to overuse and development of AMR (antimicrobial resistance) |
|  | Inadequate policies | Though a National Drug Policy regulates the use of all medicines in Ghana, the provisions in the policy do not provide for control of antibiotic resistance and use. |
|  |  | Good policy is not enough to sustain reductions in child mortality; reliable supply and access to effective essential drugs are critical. |
|  |  | Pharmaceutical representatives claim that existing policies prohibit them from supplying prescription-only medications to patent medicine vendors (PMVs) who lack the requisite training and authorization to dispense such medications. |
|  | Inadequate procedures | The procedures from that law were too difficult for the essential supply of medicines in the hospital, took too long, and did not respect the principle of emergency that conditioned the hospital pharmacy medicines management. |
|  |  | Medicines available elsewhere in suitable dosage forms had not, at the time of the survey, been registered by the local medicine's regulatory agency; and so were not available in Nigeria as licensed products. |
|  |  | Staff at health care facilities were asked to report drug quality problems. Still, when there was limited feedback, or worse when the supplier was awarded the tender again, it was challenging to remain motivated. |
|  | Lack of price regulations | The absence of a clear medicine pricing policy, high retail markups, high variation in prices of medicines and the lack of a system of pharmaceutical evaluation influenced to raise pricing of drugs in the health facilities of Ethiopia |
|  |  | Median prices were several folds higher than the international reference prices (IRP), especially in the non-public sectors. |
|  |  | Most medicines were sold at markedly higher prices than their international reference prices. |
|  |  | Median prices were several folds higher than the IRP, especially in the non-public sectors. |
|  |  | The lack of effective pricing policies and regulations means prices in the private sector pharmacies will remain higher than international prices. |
|  |  | Even with low-cost, low-cost, essential medicines for non-communicable diseases, essential medicines are still unavailable in most low- and medium-income countries, partly attributed to the high levies and taxes that are sometimes imposed on medicines and the uncontrolled markups by distributors and retailers. |
|  |  | Cardiovascular medicines for oral use were more expensive than the International Reference Prices and recommended list prices in the Federal Health Insurance Scheme in the study locations. |
|  |  | Most medicines were sold at markedly higher prices than their international reference prices. |
|  |  | The chemotherapy pricing market is highly variable and is not transparent for reasons that remain unclear |
|  |  | Drug prices are not controlled/regulated in Ethiopia, and there is no agreed-upon method or legally enforceable mechanism to determine the final. |
|  |  | No national medicine pricing policy exists for the private sector; for patients covered by the Medical Insurance Scheme of RSSB, this insurance allows a maximum profit margin of 40% over the private wholesalers' prices. |
|  | Lack of pharmaceuticals evaluation system | The absence of a pharmaceutical evaluation system influenced the raised pricing of drugs in the health facilities of Ethiopia. |
|  | Lack of political will | Lack of political will in implementing policies. |
|  | Lack of regulatory body | Lack of regulatory bodies for certifying and professionalizing medical logistics companies. |
|  | Lack of structured regulations | Results from this study also seem to suggest that Nigeria need to develop specific guidelines for registration and control |
|  |  | One of the major weaknesses in today's health sector is the non-existence of important health legislation and the outdatedness, contradictions, and ambiguities of some existing health policies. |
|  | Lack of policies | Lack of inclusion in checklists and lack of a written policy were barriers to the routine use of capnography during procedural sedation. |
|  |  | The general view from the paediatricians was that IMCI does not cater for child-friendly medicines due to the lack of a conducive medicine policy environment. |
|  | Long registration process | One manufacturer estimated that registration time increased from 3 to 6 months to one year and noted that the assessment was becoming more stringent. |
|  |  | Delayed process of medicine registration. It takes 2-3 years and has high expenses. |
|  |  | Regulations were considered a barrier. It caused extra work, requiring additional documentation books and special licenses for prescribing opioids. |
|  | Incompliance to regulations | Contrary to current regulations, most shops offered amoxicillin, and those stocking zinc were minimal. |
|  |  | The other contradiction is that despite the absence of dispersible tablets in the EMHSLU, development partners have been distributing them in the community, bypassing government health facilities |
|  |  | Our findings suggest a large variation in policy adherence, and each facility decides whether to charge for the medicines dispensed. |
|  |  | In addition to medicines purchased from drug shops in Tanzania, 31% were not licensed for sale in such shops, notably antibiotics, second-line antimalarials, metronidazole and diclofenac. |
|  |  | Notably, none of the samples collected in CHAM facilities, and only one of the samples collected in public health facilities, failed quality testing. |
|  |  | Unregistered products were found in surveyed drug shops and pharmacies |
|  | Restriction on use of medicines | The use of some in-formulary medicines might have restrictions attached to them. |
|  |  | While the WHO protocol recommends antibiotics for pregnant women who give birth, the National Health Insurance Drug lists for CHPS compounds prohibit the prescription of antibiotics to newly delivered mothers, despite it being mandatory. |
| Donor policies | Donor's influence   - Supply of unneeded medicines - Lack of donor priority - No systematic registrations | The clarity in the relationships between global funders (e.g., PEPFAR and Global Fund) and national and provincial health ministries as to which entity is fiscally responsible for which operational or technical task. |
|  |  | ICEMs were not a donor priority. Donor funding might help improve access. |
|  |  | Policies of the major international funders of medicines procurement for specific diseases, particularly HIV/AIDS and TB, present a sharp contrast. PEPFAR has set up new regional distribution centres in Ghana and Kenya for HIV medicines. |
|  |  | Donations are received irrespective of need. |
|  |  | Certain importers of donor-funded goods do not systematically register the import requests with ZAMRA. |
|  | International procurement policies | International procurement policies of donors and NGOs disadvantage local producers if they have neither WHO prequalification nor approval from a well-established authority. |
|  | Policy adoption | Failure to officially roll out the policy from the global to the national level grossly affected the level of awareness that the stakeholders have about child-appropriate dosage formulations. |
| Influential people | International recommendations | The international community does not back up local pharmaceutical production, hence relying on importations. |
|  |  | International recommendations. Informants said Tanzania has vertical programmes for HIV/AIDS, malaria, tuberculosis and leprosy. These programmes have their guidelines, which are updated based on global recommendations. These recommendations were adopted in the STG |
|  | Limited health managers influence | While these nurse managers demonstrated situation-based performance to minimize the impact of low staffing levels on the quality and safety of patient care, they had less influence on policy and resource development to ensure incentives and timely payment for nurses to improve their motivation to work and reduce absenteeism. |
|  | Donor's influence | This dual arrangement reflects the policy-practice gap, whereby donors operating outside the policy framework have introduced child-appropriate dosage formulations and selected their channels for distributing these medicines in the community. |
|  | Pharmaceutical's promotion | Promotion of medicines by the pharmaceutical industry. Some informants accused medicine promotion of influencing prescription practices. They were concerned that the medication recommended for addition to the STG was there because of these influences.  In the informal interviews, drug representatives acknowledged that they persuade healthcare workers to procure and prescribe their products. They give them free medicine samples and gifts such as stationery, refrigerators, and televisions. |
| Corruption | Discrimination of health workers | Discrimination was, however, reported that some of the counties rejected the doctors sent to them because they had come from different tribes or counties from the ones they were posted to. |
|  | Corrupt practices | Corruption in public companies |
|  |  | Participants pointed out that tenders were sometimes given to companies that could not deliver, which has important implications for the patient care within their hospitals |
|  |  | Financial incentives were used to ensure pharmacists dispensed specific generics within their chain of pharmacies. |
|  | Counterfeit trading | A concern with availability might be the problem of counterfeited drug markets in developing countries. |
| Political stability | Political unrest | The geographical terrain and political unrest also impacted the numbers of nursing staff employed in these rural hospitals |
