# Supplementary material for: Implementing essential diagnostics-learning from essential medicines: A scoping review
Source: PLOS Glob Public Health. 2022 Dec 19;2(12):e0000827. doi: 10.1371/journal.pgph.0000827 (PMC10121180; doi:10.1371/journal.pgph.0000827)
Supplement: S2 Table — (DOCX) [file pgph.0000827.s003.docx]

**Enablers to the implementation of Essential In Vitro Diagnostic List (EDL)**

| **Subthemes** | **Codes** | **Illustrative quotes** |
| --- | --- | --- |
| **Theme: Level – Health system** | | |
| Accessibility of care | Affordability of tests | The biochemical test price is the lowest in each hospital, using the median relative price of the five hospitals. |
|  | Geographical accessibility | An urban lab had a trending association with higher EDL availability. |
| Facilities | Availability of on-site laboratories | All RRHs and GHs (100%) and 12 of the HCIVs (86%) had an on-site laboratory. |
|  | Availability of tests | The availability of diagnostics (blood sugar test, urine dipstick-protein test, and urine dipstick-ketones test) was 96%. |
|  |  | As a gold standard malaria test, Microscopy was found to be available in all selected hospitals. |
|  |  | Rapid diagnostic test (RDT) as an alternative for Microscopy was available in 75% (15/20) of hospitals |
|  | Facility capacity | All clinical laboratories have the infrastructure to provide basic services. |
| Procurement and distribution systems | Good inventory levels of tests | Inventory levels of Determine test kits (HIV) were reported as being at adequate levels. |
| Relationship with norms and standards | Availability of guidelines | Availability of guidelines for the diagnosis and treatment of HTN and  diabetes was 94%. |

**Enablers to the implementation of the Essential Medicines Lists (EML)**

| **Subthemes** | **Codes** | **Illustrative quotes** |
| --- | --- | --- |
| **Theme 1: Level – Recipients of Care** | | |
| Knowledge and skills | Adequate knowledge | Patients with little education and wealthier were likely to find the medicines they needed. |
|  |  | Once participants understood that they had an NCD, they described taking their health seriously and viewed medicines as a priority. |
|  |  | 95.8% of the patients knew five basic WHO drug use indicators at Auditable pharmaceuticals service and transaction system (APTS), and 91.6% of patients in non-APTS sites. |
|  |  | Most patients with uncomplicated malaria (78%) demonstrated correct knowledge regarding artemether-lumefantrine (AL) dosing, including the amount of drug per dose, the number of doses per day and the total number of days to complete the full dose for patients who received AL. |
|  | The desire for increased knowledge | Community women noted that access to Maternal, Newborn, and Child Health (MNCH) services increased due to newly constructed and upgraded healthcare facilities in underserved areas and expanded service provision. |
|  | Knowledge of diseases | Participants knew how to deal with the health problem and took self-treatment. |
| Motivation to change or adopt a new behaviour | Accessibility to the health facility | Patients preferred to be treated in a nearby health facility. |
|  |  | The ease of walking to a health facility without worrying about the availability of transport was described as a facilitating factor. |
|  | Adequate healthcare support | The facility's reputation, availability of doctors, clean environment, and shorter waiting rooms. |
|  |  | Additional motivating factors included the perceived quality of care and education they had received when attending antenatal care. |
|  |  | Across all study sites, women were prepared for Option B +through health education and counselling. |
|  |  | The availability of skilled personnel who can conduct lifesaving interventions such as caesarean section, blood transfusion, labour induction and infusion was considered a reason to deliver in a health facility. |
|  |  | Availability of birth notification, drugs and other commodities given to women after delivery, such as diapers, towels, basins and mosquito nets, also motivated women to deliver in a health facility. |
|  |  | Health education/advice received during antenatal clinics, home visits or seminars encouraged women to deliver in a health facility. |
|  |  | The patient satisfaction survey results revealed a high level of patient satisfaction. |
|  |  | All women agreed that the services offered and supported by the NFHISG insurance scheme essentially met their needs and expectations. |
|  |  | Improved patient-provider interactions, measured by patient perceptions of provider kindness during deliveries |
|  |  | Availability of drugs and supplies, health workers, communication, type of health care provided, and attitude of health workers were significantly associated with overall client satisfaction. |
|  |  | Study participants noted that mothers were pleased to take a single ARV tablet daily compared to other HIV-positive patients who took more than one pill daily. |
|  | Accessibility of health facility   - Availability of medicines | Patients stated that their reason for purchasing drugs from Jimma Health Centre pharmacy was availability. |
|  |  | The hospital's full and partial drug availability was also highly preferred by respondents in the current study. |
|  |  | 82.4% of the patients were satisfied with the availability of prescribed drugs in APTs hospitals compared to 73% in non-APTs hospitals |
|  |  | Availability of essential drugs. |
|  | Adequate financial access   - Affordable medicines | Patients stated that their reason for purchasing drugs from Jimma Health Centre pharmacy was the cheap price. |
|  | Existing chronic disease | Having an existing health condition, such as high blood pressure, iron deficiency, or being sick, played a big role in motivating them to deliver in a health facility. |
|  | Social welfare support | Many informants associated patients' choice of a health care facility with its closeness to other services such as social grant collection points and shopping centres. Providers reported that many patients rely on their or a family member‚ social grant to cover transport expenses. |
| **Theme 2: Level – Providers of care** | | |
| Knowledge and skills | Adequate knowledge | Participants reported that key PMTCT health providers were trained before their involvement in the provision of Option B+ |
|  |  | Staff were trained in malaria diagnosis and treatment. |
|  |  | Adequate training on malaria management in urban areas. |
|  |  | All pharmacists know that the essential list of drugs should be available in the facility according to the common health problems in the community, and MOH procures this list through bids. They choose the least price offered by companies. |
|  |  | Nearly all PHCs had sufficient capacity for screening, and most had the capacity for diagnosis and confirmation of hypertension. |
|  | Adequate provider skills | Half of the respondents agreed that the personnel who dispense essential medicines in the public health facilities under the hybrid pull and push supply system are adequate and able to manage the medication well. |
| Attitudes regarding programme acceptability, appropriateness, and credibility | Quality of medicine | The healthcare providers all felt that the quality of medicines in South Africa was good. |
|  |  | The quality of drugs depends on the manufacturing company. |
|  | Positive attitude to ICT | There was generally a positive attitude to computers and the Internet for educational matters; everyone saw a possibility to improve their medical knowledge and skill. |
|  | Satisfaction with social health insurance provision | Current coverage provided by the social health insurance scheme is a positive evolution. |
| Motivation to change or adopt a new behaviour | Evidence approach | The ability to obtain expert opinion influenced care decision-making. |
|  |  | The two informants who mentioned being trained in evidence-informed decision-making said their recommendations were supported by research evidence from clinical trials and meta-analyses. |
|  | A positive interest in ICT | All participants stated that they were interested in computers and very interested in improving their ICT skills. |
|  | Supportive supervision | In support of the official policy, districts undertook activities that increased the involvement in or awareness of the community case management (CCM) of childhood illness programs among health centre staff. |
| **Theme 3: Level – Other stakeholders (community health committees, community leaders, programme managers, donors, policymakers, opinion leaders** | | |
| Knowledge and skills | Adequate knowledge | Medicine's quality, safety, and efficacy are considered first, followed by cost considerations, including pharmacoeconomic evaluations and drug pricing during essential medicine list (EML) selection. |
| **Theme 4: Level – Health system facilitators** | | |
| Accessibility of care | Access to health facilities   - Adequate geographical access - Free care - Short waiting times - Access to medicines | Urban health facilities were associated with a statistically important reduction in malaria mortality compared to those in rural areas. |
|  |  | Access to MNCH services increased due to newly constructed and upgraded healthcare facilities in underserved areas and expanded service provision. |
|  |  | A hospital with less waiting time was preferred over long waiting times. |
|  |  | The number of visits in the previous three months was significantly higher in the rural group than in the urban group. |
|  |  | In the capital city of Maputo, anaesthesiologists report access to all these medications |
|  |  | Locally produced medicines are more accessible than imports for rural consumers, and medicines are both imported. |
|  | Adequate financial access   - Affordable medicines - Lower drug prices - Free care | Health care financing enabled clients to avail free delivery services at health institutions, especially at public health centres. |
|  |  | Parenteral benzathine penicillin, oral furosemide, glibenclamide, bendrofluazide, atenolol, cardiac aspirin, digoxin, metformin, captopril and nifedipine were the only affordable drugs. |
|  |  | NHIF's price for pain was lower than the pharmacy price in all regions. NHIF price for CVD drugs was lower than pharmacy price in all regions. NHIF's price for diabetes drugs was lower than the pharmacy's price. |
|  |  | All the medicines listed were affordable. |
|  |  | The affordability of LPGs was generally suitable for all medicines, with standard treatment costing. |
|  |  | Commonly used oral antibiotics for adults and children were mostly affordable. |
|  |  | The private sector reported limited availability of drugs surveyed, suggesting the free provision of HIV, Malaria and TB in public facilities. |
|  |  | HIV/AIDS and tuberculosis are distributed free of charge. |
| Financial resources | Sufficient funding   - Access to credit financing - External funds - Insurance funds | Access to credit in the form of stock from a supplier in the past six months. Most outlets with access to stock credit from their medicine supplier were rural. |
|  |  | The Federal MOH and UNICEF mobilised external resources to procure and distribute commodities from different sources. |
|  |  | Multiple stakeholders contributed to the program rollout and enhancement |
|  |  | Since establishing the revolving drug fund (RDF), the fund's capital was always sufficient to ensure an uninterrupted supply of asthma medicines in the five pilot sites. |
|  |  | All three facilities have demonstrated profitable operations after one year of operation after utilisation of the Revolving Fund Pharmacy model (RFP) |
|  |  | After NHIS internally generated funds increased. |
|  |  | Support from social insurance schemes. All women agreed that the services offered and supported by the NFHISG insurance scheme essentially meet their needs and expectations. |
|  | Financial autonomy | The financial autonomy resulting from bank accounts enabled facilities to use bonus funds and cost-sharing revenue (from user fees and community-based insurance) to procure drugs and supplies. |
| Human resources | Adequate capacity   - Availability of health workers | The average staff availability was higher in peripheral health centres than in medical centres. |
|  |  | Most PHCs had sufficient human resource capacity according to the self-report of two or more full-time staff at data collection. |
|  |  | Availability of an assistant during procedural sedation. |
|  |  | The availability of Human Resources health among the facilities increased during the Integrated Supportive Supervision (ISS) visits. |
|  |  | All clinics had both professional and enrolled nurses, and all had a consulting doctor for four or more hours per week for all complicated cases. |
|  |  | Model B allowed for centralising a greater portion of the supply chain functions within MSL, a specialist supply chain organisation than removing some of the burdens from the non-specialist staff at the District Health Offices. |
|  |  | The increased human resources, decentralised drug distribution systems and robust data collection systems enabled the MOHS to provide approximately 80% of medications. |
|  |  | After implementing the ophthalmic health system Strengthening (HSS package), there was a significant increase in the proportion of intervention sites with a staff member. |
|  | Adequate on-the-job training | More than 80% of them had their staff trained and knew the guidelines for malaria management. |
|  |  | Most participants felt that the training they had received was adequate. |
|  |  | All health surveillance assistants (HSAs) reported receiving community case management (CCM) training. |
|  | Complementing health workforce | The accredited drug dispensing outlet (ADDO) program not only fills a human resource niche in the health system. |
| Education system | Access to training   - Establishment of training institutions | Stakeholders in Tanzania identified the institutionalisation of training of the dispensers as a critical step toward independence from donor funding. In preparation for public and private training institutions |
|  |  | The ADDO training will now be conducted exclusively through approved training institutions |
|  | Training of health workers | Pharmacy assistant training and deployment to rural health centres in Malawi increased access to antimalarial medications for children under five years in the short term.  The Village Reach PA training program expanded the health workforce in Malawi, and the deployment of PAs appeared to increase community access to antimalarials in the short term. |
|  |  | P.A training led to increased data quality and improved adherence to storeroom management and dispensing standards. |
| Clinical supervision | Regular supportive supervision | The key informant interview results showed that three-fourths of the health posts received weekly follow-ups and supervision. |
|  |  | Participants noted that supervision and mentorship took place on different dates, and other teams did it to enhance their performance. |
|  |  | District managers do supportive supervision and cascade coaching by peer health facility personnel. |
|  |  | The verification system under pay for performance (P4P) also intensified district supervision, allowing district managers to identify and address stock-outs of a wider range of drugs. |
|  |  | Improved timeliness of supervision, associated with the verification activities carried out as part of the P4P programme, significantly mediated 15% of the effect of P4P on the uptake of two doses of antimalarials during pregnancy |
|  | Peer cascade coaching | Through peer learning, staff confidence increased, and knowledge improved, leading to better understanding and compliance with Jazia's prime vendor system (PVS) operation. |
|  |  | The nurses who had performance issues were paired with experts or role models to allow them to learn. |
|  |  | The practitioner-researcher shared research skills, such as learning while doing, with the health workers at the individual primary care facilities. |
| Internal communication | Good coordination | There are guidance from the district pharmacy on ensuring that drug stock levels do not fall below the minimum or emergency- stock level thresholds. |
|  |  | Frequent contact between district managers and providers may also help reduce stock-outs. |
|  |  | The direct flow of demand information from the health facility to medical store limited (MSL) (parastatal agency) and pre-packaged supplies for the health facility were the key performance-enhancing attributes of Model B. |
|  |  | Frontline health workers received regular updates on the stock situation from the depots, which enabled them to make alternative arrangements where possible. |
| External communication | Use of community peer educators | The use of peer educators to provide targeted education to the community appeared to have impacted the increase in patient numbers. |
|  | Good communication practices | Facilities that review clients' opinions were more likely to be ready to provide basic emergency obstetric and newborn care (BEmONC) services. |
|  |  | The survey result showed that all of the respondents knew the HEWs, and the majority of the mothers received advice during their recent pregnancies and in the postpartum period. |
|  |  | Nurse managers also identified that engaging patients or their family carers in quality improvement were of great importance |
|  |  | Evidence shows that communication between the client and the health care provider significantly impacts the client's satisfaction. |
|  |  | Good physician and nursing communication were among the most important characteristics of health care services preferred by respondents. |
|  |  | Medicine packets include information in Kiswahili, increasing accessibility and trust for purchasers. |
|  |  | HSAs counselled caregivers about the dose, frequency and duration of treatment for over half of children who provided ORS, antibiotics or antimalarials (61%), and 81% of caregivers described how to give these treatments correctly. |
| Allocation of authority | Financial autonomy | Implementing a direct health financing facility (DHFF) was an important prerequisite for the Jazia prime vendor system (PVS) to be successfully implemented because facilities have financial autonomy and flexibility in using funds held in their bank accounts. |
| Accountability | Structured system | Accountability activities of the Jazia prime vendor system (PVS) are anchored within the regional and district health administration structures for sustainability purposes, and no new or parallel structures were created for implementation. |
|  | Adequate tracking/Management system | APTS hospitals had a daily sales tracking/management system |
|  |  | The direct flow of demand and order information from health facilities to MSL under Model B reduced the problem of diffuse accountability. |
| Management and/or leadership | Adequate leadership | Strong leadership and commitment by the Federal Ministry of Health (FMOH) and pharmaceuticals funds and supply agency (PFSA) to ensuring sustained supplies have been critical to launching a new national initiative. |
|  |  | Managerial interventions were also instrumental in promoting the use of generic medicines. |
|  | Facility leadership | Support supervision is good and adequate in the public health facilities of Gulu District. |
| Information systems | Good information management practices | Dispensers' records at ADDOs have allowed supervision and inspection teams to review and assess ADDO dispensers‚ performance and the shops‚ compliance with regulations. |
|  |  | The implemented stock card system improved organisation and inventory tracking. |
|  |  | At the national level, the forecasts of the demand for selected medicines are based on the data collected by the HIS (or MTUHA, as known in Tanzania). |
|  |  | The direct flow of demand information from the health facility to MSL and pre-packaged supplies for health facilities were the key performance-enhancing attributes of Model B. |
|  |  | The verification system under P4P provided more opportunities for district managers to identify and address stock-outs of a wider range of drugs. |
|  |  | Pharmacy assistants training led to increased data quality and improved adherence to storeroom management and dispensing standards. |
|  | Access to record-keeping tools | All health posts had integrated community case management (ICCM) registration books, ICCM chart booklets, integrated maternal and newborn service cards, and community-based newborn care (CBNC) key indicator monitoring charts. |
|  | Utilisation of information communication technology | There is a computer program to calculate the quantities needed for each facility. |
|  |  | A digital tool electronic logistics management information system (eLMIS) gives the district pharmacy and the Ministry of Health to monitor real-time data on health facility NCD drug consumption and stock levels more regularly. |
| Facilities | Adequate capacity | Nearly all PHCs had sufficient capacity for screening, and most had capacity for diagnosis and confirmation of hypertension. |
|  | Availability of medicines | Among the 15 investigated public facilities, the availability of the antimalarials artemether/ lumefantrine tablets and sulfadoxine/pyrimethamine tablets was 93% in the facilities of the Christian Health Association of Malawi (CHAM), even 100% of. |
|  |  | Calcium channel blockers and insulin were the only medication most likely available to manage NCD in urban and semi-urban compared to rural facilities. |
|  |  | Private sectors were better at having essential medicines compared to their encounters. |
|  |  | Pharmacies generally reported higher availability rates for all five essential medicines assessed compared to drug shops. |
|  |  | All participants pointed out that their health facilities had never run out of ARV medications for women. |
|  |  | Among the key performance indicators of Jazia PVS are increased availability of essential medicines at the facility and product delivery time at the district headquarter. |
|  |  | The study showed that all facilities improved the availability of essential medicines after introducing the NHIS compared to before the NHIS. |
|  |  | Higher availability of antibiotics in public pharmacies as compared with private ones. |
|  |  | Study findings showed a wide variety of fixed-dose combinations (FDCs) available in the study areas. |
|  |  | Both the originator brands and generic equivalents were found in the private pharmacies. The availability of OB medicines was higher in the public sector versus the private sector. |
|  |  | Locally produced medicines are more accessible than imports for rural consumers. That medicines are both imported and locally produced display greater rural and urban availability than those which are import-only. |
|  |  | All informants said that the medicines they select must be available on the Tanzanian market so that patients can access them even when they are not available at public healthcare facilities. |
|  |  | HPs did not have much shortage of essential commodities, as implementing partners were providing the supplies directly to zones and districts in parallel to the existing system |
|  |  | Ninety-six (91%) HFs had either the first line (AL) or second line (artesunate-amodiaquine (ASAQ)) ACT available for the entire day of the survey.  Nearly all HFs (98%) had at least one injectable pre-referral treatment in stock. |
|  |  | Varying medicine stock-outs are experienced at the PHC facilities, with the health centres having better availability than the clinics. |
|  |  | In only 10% of the clinics, clinic managers reported no functional stock-outs of essential drugs and nutritional supplements.  Antiretroviral drugs (zidovudine and nevirapine) were available in 19 clinics (95%) as part of the programme for the prevention of mother-to-child transmission (PMTCT) of HIV. |
|  |  | Medicines availability was higher in the urban informal sector, where up to 63.6% of the medicines were available. |
|  |  | All the public primary schools had first aid boxes and essential drugs and materials. Among 50 (94.3%) private schools with first aid boxes, 47 (88.7%) had essential medicines and materials. |
|  |  | Medicines were found more frequently in private pharmacies compared to public pharmacies.  Medicines were provided free at seven public hospital pharmacies nationwide. |
|  | Improved infrastructure | Expanded hospital warehouse, built shelves, implemented a stock card system, improved organisation and inventory tracking |
|  |  | We installed emergency cabinets on the wards to provide essential medications and supplies during hours when the pharmacies were closed. |
| Patient flow processes | Good referral practice | Seventeen per cent of patients were referred to the hospital through a formal medical channel. |
|  | Community-based health care | CHWs and CHEWs improve continuity of care by strengthening the health referral system from the community to the facility level. |
| Procurement and distribution systems | Efficient supply management   - Improved supply management - Innovative systems - Utilisation of ICT | HPs did not have much shortage of essential commodities, as implementing partners provided the supplies directly to zones and districts in parallel to the existing system. |
|  |  | The data show that the market supplies sufficient products to treat all suspected and unconfirmed cases. |
|  |  | Our findings indicate an overall positive impact on both equipment maintenance and drug stocks because of the incentives provided through the scheme. Direct district level involvement not only improved supply management at intervention facilities. |
|  |  | Enough supplies were made available at the national level to respond to replenishment requests. Overstock and understock problems in HPs were corrected through redistribution among HPs by district staff. |
|  |  | ARV medication had reduced supply, indicating that supply chain management had improved over time |
|  |  | Across facilities and districts in the two regions, the study participants felt that the prime vendor system (PVS) had increased the availability of medicines at the facilities. |
|  |  | The tool revolving drug fund (RDF) for managing medicine ordering was useful in estimating the number of months covered by inhalers in stock and the approximate date when the next medicine order should be placed. |
|  |  | The verification system under P4P provided more opportunities for district managers to identify and address stock-outs of a wider range of drugs. |
|  |  | The short-dated stock report generated by the RxSolution system helped the hospital redistribute stock to other hospitals that needed the supply. They were able to utilise these medicines before they expired. This helped minimise waste in this hospital and improve medicines availability in other hospitals. |
|  | Lower public procurement costs | Public hospitals procure medicines through MSD below the available international market. |
|  | Regular distribution systems | Essential drug kits are distributed to governmental HF by the DMO's Office on a regular quarterly (and sometimes monthly) basis. For this reason, SP is rarely out of stock in governmental (public). |
|  | Good store management practices | There is adequate store management, particularly stores practices first expiry first out. |
|  |  | Model B facilities exhibit significantly higher commodities rates stored and organised according to First to Expire, First Out (FEFO) principles. Storage is kept at the appropriate temperature, appropriate fire safety, and products are stored on pallets and shelves. |
|  | Good inventory management | Pharmacy technicians-maintained stock lists, which the task force evaluated monthly and used for forecasting, allowing the Koidu Government Hospital and Partners in Health teams to anticipate needs and procure appropriately. |
| Incentives | Mentorship | Mutual learning partnership among healthcare facility staff to help them unfold best practices and capabilities/creativity to address health facility challenges to improve service delivery. |
|  | Financial incentives to health facilities and managers | When the National Health Insurance Fund incorporated ADDOs into its scheme, owners felt that the arrangement boosted their sales. |
|  |  | Our findings indicate an overall positive impact on both equipment maintenance and drug stocks because of the incentives provided through the scheme. |
|  |  | Financial incentives were used to ensure pharmacists within their chain of pharmacies dispensed specific generics |
|  |  | By providing incentives to facilities and districts, the scheme ensured that stakeholders at all levels were working towards the same goals |
|  |  | District managers are rewarded for reducing the proportion of facilities reporting stock-outs of essential medicines in the district. |
|  |  | Stock visibilities are higher in Models A and B due to clear incentives for reporting stock, consumption, and receipt data to higher levels in the supply chain. |
| Relationship with norms and standards | Availability of guidelines | Most caregivers reported the existence of national guidelines. |
|  | Compliant to EML | Almost all pharmacists in the rural unit (90-100%) are prescribed from the essential drug list and are instructed not to prescribe from outside the list. |
|  | Compliant to guidelines | Almost none of the PHUs in the Bombali district were successfully providing all the critical BEmONC services as defined by WHO |
|  |  | Midwives methodically follow a treatment protocol and emphasise the importance of antenatal visits and other steps during intrapartum. |
|  | Compliant to policies | It was evident that NIM-ART nurses followed the national ARV policies when initiating and maintaining ART. |
|  | Compliant with standard operating procedures | In all healthcare facilities surveyed, it was reported that they do comply with the Standard Operating Procedures. |
|  | The rationale for the use of drugs | The percentage of antibiotic encounters among the prescriptions was 5.11%, within the WHO recommended standard of less than 30%. |
| **Theme 5: Level – Social and Political facilitators** | | |
| Legislations or regulations | Structured registration | For any medicine to be allowed to enter the Tanzanian market, it has to go through a rigorous registration process in which its quality, efficacy and safety are thoroughly checked. |
|  | Supportive policies | The Jazia PVS implementation supported health financing policy reforms in the study districts. |
| Donor policies | Donor's influence | Our findings show the success of the Malawi government, the faith-based organisations and the donors in making these antimalarial medicines widely available in Malawi. |
| Influential people | Government agency | In Tanzania, the wholesale-to-retail market was strongly influenced by the market weight of the government buying agency. |
|  | International organisations | The WHO national programme officer for Essential Drugs and Medicines provided technical guidelines on global best practices and lessons from other health systems and guidance on the WHO model list of medicines and the WHO classification of antimicrobial. |
