# Supplementary material for: Implementing essential diagnostics-learning from essential medicines: A scoping review
Source: PLOS Glob Public Health. 2022 Dec 19;2(12):e0000827. doi: 10.1371/journal.pgph.0000827 (PMC10121180; doi:10.1371/journal.pgph.0000827)
Supplement: Attachment — Submitted filename: Response to Reviewers.docx [file pgph.0000827.s007.docx]

Moriasi Nyanchoka

Kenya Medical Research Institute - Centre Global for Health Research

P.O Box 20778-00202,

Kisumu, Kenya

Email: [Moriasi.nyanchoka@outlook.com](mailto:Moriasi.nyanchoka@outlook.com)

31 October 2022

The Editor,

PLOS Global Public Health.

Dear Editor,

**RE: RESPONSES TO EDITOR'S AND REVIEWER'S COMMENTS AND PROPOSITIONS**

Please find enclosed our revised manuscript titled “Implementing essential diagnostics-learning from essential medicines: A scoping review” for review by PLOS Global Public Health. We thank you and the reviewers for your constructive comments on our manuscript. We present a point-by-point response to the peer review comments below. We believe we have sufficiently addressed the peer review and trust that it may be accepted for publication.

The line numbers used below to highlight changes are based on the ones in the revised manuscript (marked-up copy).

**Editors Comments:**

| **Editors Comment** | **Author response** |
| --- | --- |
| Competing interests | We have updated the competing interests statement in the manuscript—changes in line 1156. |
| Figures resolution | We have made revisions. We re-uploaded all the figures to the PACE platform to improve the resolution |
| The objective of the scoping review | The objective stated was in line with the guidance in the JBI manual. Scoping reviews aim to provide an overview or map of the evidence.  A scoping review of scoping reviews found that the three most common reasons for conducting a scoping review were to explore the breadth or extent of the literature, map and summarize the evidence, and inform future research (Tricco et al. 2016b)  The value of scoping reviews to evidence-based healthcare and practice lies in the examination of a broader area to identify gaps in the research knowledge base (Crilly et al. 2009, clarify key concepts (de Chavez et al. 2005), and report on the types of evidence that address and inform practice in the field (Decaria et al. 2012)  The scoping review question guides and directs the development of the specific inclusion criteria for the scoping review: The question we asked was “What evidence is available on the implementation of essential lists in Africa to guide the implementation of the newly introduced EDL?”  Our objective was therefore to map evidence on the implementation and evaluation of the WHO’s essential lists in African countries to guide the effective implementation of the new WHO EDL.  We have revised our objective to *map evidence on the implementation and evaluation of the WHO’s essential lists in African countries to guide the effective implementation of the new WHO EDL.*  Changes are in lines 146 to148. |
| Description of how the review thematic analysis was conducted | We have made changes. We have provided details on how we conducted thematic analysis in the data extraction and charting section on lines 300 to 304. |
| Quality check in a scoping review | Critical appraisal is not mandatory; however, reviewers may decide to assess and report the risk of bias in scoping reviews depending on the purpose of the review.  For our review, mapping the studies and designs used to assess the evidence on implementing the adopted WHO lists is one of our outcomes (line 216). In addition, we also aimed to highlight limitations and methodological gaps in the type or designs used to appraise implementation and intervention programmes within the field of essential diagnostics and make recommendations for future research. Thus, the critical appraisal was key to our scoping review. |
| Aligning the article with the reporting guidelines - PRISMA-ScR | We have revised the flow of the results section as guided by the PRISMA-ScR checklist. Changes in line number 177 – 217 and 400 - 405. |
| Presentation of results of individual sources of evidence | Due to the number of studies included in our scoping review, we have presented each study's key findings as a supplementary file (S3 Appendix). However, we have indicated the individual study summary in lines 368 to 369. |
| Length of the article | We have revised the manuscript and shortened the length to 5,886 words from 8,385 words.  We have focused the results section on the main- barriers and enablers in all the domains (health systems, social and political levels, providers of care, recipients of care and other stakeholders) as raised by reviewers 1& 2.  We have omitted table 5 on qualitative assessment since we have provided the details in the text and S3 Appendix. |

**Peer Reviewers Comments**

| **Reviewer 1** |  |
| --- | --- |
| **Peer review comments** | **Author response** |
| Length of the article | We have revised and shortened the length of the manuscript to 5,886 words from 8,385. Extra information such as has been moved to supplemental material.  Changes are in line numbers 406-409, 430-465, and 477-872. |
| EDL and EML – Implementation. How many African countries have national EML and EDL based on WHO guidance? | We have incorporated the number of countries that have adopted and implemented WHO essential lists. Changes are in lines 1024 -1030.  Our review mapped evidence on the implementation of adopted WHO model lists (EDL and EML) into national essential diagnostics and medicines lists (NEDLs and NEMLs). It looks beyond adopting the WHO essential lists since the national lists alone would have no or less impact on health outcomes. An enabling environment is crucial in implementing National lists and, subsequently, the effect on the health outcomes of populations. We drew from these barriers and enablers to inform further the adoption and implementation of the newly developed WHO model list of EDL and impact health outcomes, responsiveness, social and financial risk protection and efficiency, as envisioned. |
| **Reviewer 2** |  |
| **Peer review comments** | **Author response** |
| Repetitive result section: Similarity in individual, health system and policy level (HR, lack of knowledge, lack of training and guideline knowledge). | We have revised the results section and focused on the most common barriers reported in each domain (health systems, social and political levels, recipient of care, provider of care and other stakeholders). The revisions have improved the flow of the presented results and addressed repetition.  Changes are in line number 873 – 944. |
| Restructure the discussion to focus on the key findings. Evaluate differences between countries which have implemented EDL – Nigeria | We have summarised the key findings of our review in lines numbers 967-953, 1000-1022, and 1010-1022.    We further discussed strategies and critical considerations crucial to improving their impacts on the health outcomes of the populations, responsiveness, efficiency, and social and financial risk protection since the health systems' blocks are interconnected and related and do not stand independently. Health service significantly depends on all other building blocks of the health system. These are discussed in lines numbers 1044 – 1062.  Though the evaluation of differences between countries and the implemented EDL for Nigeria would be valuable, it is beyond the scope of our review. It would also be impractical since it's the only African nation to publish its national EDL, and it could be because WHO recently introduced the Model list for EDL in 2018. Additionally, different countries could be in the process of developing their own national EDLs. |
| Described countries in regions | We have presented the countries in regions to highlight the origin of articles per region. Highlighting single countries in tables 1 and 2 will lengthen the tables considering the number of included studies. However, each article's place of study (country) is provided in the S3 Appendix. The presentation also helps map the lack of or the number of studies/data on essential lists in different areas and could inform future research in these regions. |
| Items in tables 3 and 4 do not add to 100% | The proportions presented in tables 3 and 4 only represent the prominent themes per SURE framework codes to provide a visual summary. The inclusion of all codes will make the tables lengthy. In the result section, we have presented all the codes (sub-themes) per the theme (lines 300 -304). |
| Provide a full form of ICT at the beginning | Following the revision of the results section, this part was omitted to confine the results section to the review's main findings. |

Yours sincerely,

**Moriasi Nyanchoka**
